# Healthier dietary habits are associated with lower depression and anxiety among medical students at a private university in Lima, Peru: A cross-sectional study

**DOI:** 10.1371/journal.pone.0346062

**Published:** 2026-06-18

**Authors:** Adriana Daniel Armas, Diego S. Polanco Taipe, David R. Soriano-Moreno

**Affiliations:** School of medicine, Universidad Peruana Unión, Lima, Peru; Universidad Católica Sedes Sapientiae: Universidad Catolica Sedes Sapientiae, PERU

## Abstract

**Introduction:**

Anxiety and depression are common conditions among medical students. In recent years, numerous factors associated with these disorders have been investigated; however, evidence regarding the role of dietary habits remains limited.

**Objective:**

To evaluate the association between dietary habits and the presence of anxiety and depressive symptoms among medical students at a private university in Lima, Peru.

**Methods:**

An analytical cross-sectional study was conducted in 2025 among medical students aged 18 years and older. Dietary habits were assessed using the Healthy Eating Index, while anxiety and depression were measured using the GAD-7 and PHQ-9 scales, respectively. Poisson regression with robust variance was used to calculate prevalence ratios (PR).

**Results:**

A total of 264 students were included. Only a minority had healthy dietary habits (1.1%), and the prevalences of anxiety (34.9%) and depression (45.1%) were high. For each additional point in the Healthy Eating Index, the prevalence of anxiety decreased by 3% (aPR: 0.97; 95% CI: 0.95 to 0.98), while the prevalence of depression decreased by 2% (aPR: 0.98; 95% CI: 0.96 to 0.99).

**Conclusion:**

Dietary habits were inversely associated with the prevalence of anxiety and depression among medical students. These findings highlight the importance of promoting healthy dietary patterns as part of comprehensive strategies for the prevention and promotion of mental health in this population.

## Introduction

Mental health disorders, particularly anxiety and depression, represent a major public health problem worldwide, affecting more than 300 million people and contributing substantially to the global burden of years lived with disability [1,2]. These conditions more frequently affect adolescents and young adults, a life stage characterized by significant academic, social, and personal transitions [3]. In the Peruvian context, anxiety and depression also constitute relevant mental health concerns, university student, and especially medical students, being a particularly vulnerable population, in whom the prevalence of depression ranges from 18% to 50% and that of anxiety from 17% to 54% [4]. This higher prevalence may be explained by academic pressure, lifestyle changes, the transition to adulthood, and exposure to persistent stressors [5].

Several studies have identified multiple factors associated with the deterioration of mental health among university students, including academic stress or overload, sleep disturbances, and financial difficulties [6–9]. However, one of the less explored modifiable factors in this population is dietary habits, which are particularly relevant in the Peruvian university context, where the habitual diet has been characterized by a high intake of refined carbohydrates, fats, and ultra-processed foods, potentially negatively impacting both physical and mental health [10–12]. Emerging evidence suggests that specific dietary patterns and nutrients may play an important role in mood regulation and in the pathophysiology of mental disorders. For example, the intake of micronutrients such as omega-3 fatty acids and zinc has been shown to have neuroprotective effects, whereas high consumption of sugars and ultra-processed foods has been associated with inflammatory processes and alterations in neurotransmission systems linked to anxiety and depression [13–17].

Observational studies, as well as systematic reviews and meta-analyses, have consistently reported that unhealthy dietary habits are associated with a higher prevalence of depressive and anxiety symptoms, whereas healthy dietary patterns, characterized by greater consumption of fruits and vegetables, are linked to better mental health indicators [18–22]. However, despite the growing body of evidence, important knowledge gaps remain, particularly among university populations in middle-income countries such as Peru. Most available studies originate from European or North American contexts and do not consistently account for sociocultural specificities, local dietary patterns, or the academic environments specific to medical training in Latin America [20]. Furthermore, a considerable proportion of the evidence is derived from studies that did not adequately adjust for potential confounding variables, which may overestimate or underestimate the true magnitude of the association. This gap hinders the development of context-specific, evidence-based preventive interventions and underscores the need for analytical studies that address these methodological limitations in this vulnerable population.

Hence, the aim of the present study was to evaluate the association between dietary habits and the prevalence of anxiety and depressive symptoms among medical students at a private university in Lima, Peru.

## Methods

### Design, population and sample

An analytical cross-sectional study was conducted in 2025 among medical students at a private university in Lima, Peru (Universidad Peruana Unión). Students aged 18 years and older who were enrolled from the first to the sixth academic year, carried a full academic load, and voluntarily agreed to participate were included. Students in their seventh year (internship) and those with incomplete survey data were excluded.

A non-probabilistic convenience sampling approach was used. Sample size was calculated using Epidat version 4.2, considering an approximate population of 657 medical students from the first to the sixth year, an expected outcome prevalence of 34%, a 95% confidence level, and a 5% precision, yielding a required sample of 227 students. Assuming a 20% non-response rate, the survey was distributed to 272 students.

### Context

The study was conducted in Universidad Peruana Unión, an institution sponsored by the Seventh-day Adventist Church. The Seventh-day Adventist Church promotes a healthy lifestyle that includes predominantly plant-based and balanced dietary habits, emphasizing the consumption of fruits, vegetables, whole grains, legumes, nuts, and other plant-based foods as part of its holistic health message [23].

### Variables

Depression was measured using the Patient Health Questionnaire-9 (PHQ-9), which includes nine items based on DSM-IV criteria assessing depressive symptoms over the previous two weeks. The PHQ-9 has been previously validated in medical students in Lima, Peru, demonstrating high reliability (Cronbach’s alpha = 0.903) [24]. Total scores range from 0 to 27; each item is scored from 0 to 3, where 0 corresponds to “Not at all,” 1 to “Several days,” 2 to “More than half the days,” and 3 to “Nearly every day,” with higher scores indicating greater depressive symptom severity. A score ≥10 was used to define the presence of depression, as this cutoff demonstrates adequate sensitivity (88%) and specificity (85%) [25].

Anxiety was assessed using the GAD-7 questionnaire, which consists of seven items based on DSM-5 criteria for generalized anxiety disorder symptoms reported over the past two weeks. The GAD-7 has been validated in Spanish among Colombian medical professionals and shows high reliability (Cronbach’s alpha = 0.920) [26]. The total score ranges from 0 to 21; each item was scored from 0 to 3, where 0 corresponded to “Not at all,” 1 to “Several days,” 2 to “More than half the days,” and 3 to “Nearly every day,”. Participants were considered to have anxiety if they had a score ≥10, as this cutoff has adequate sensitivity (87%) and specificity (78%) [27].

Healthy dietary habits were assessed using the Spanish-adapted Healthy Eating Index (HEI) [28]. This instrument was selected because no HEI adaptation has been validated specifically for the Peruvian population. The Spanish-adapted HEI was considered suitable for this study because it is available in Spanish, uses broad food groups that are understandable for Peruvian students, and includes dietary domains that are broadly consistent with the Peruvian Dietary Guidelines, such as fruits, vegetables, legumes, dairy products, animal-source foods, sweets, sugar-sweetened beverages, and processed meats [29]. The instrument consists of 10 items categorized into: daily recommended food groups (items 1–4: cereals and derivatives, vegetables, fruits, dairy products); weekly recommended food groups (items 5–6: meats and legumes); non-recommended foods (items 7–9: processed meats, sweets, and sugar-sweetened beverages); and dietary variety (item 10). Each component received a score from 0 to 10, where 10 indicated full adherence to the recommendations established by the Spanish Society of Community Nutrition. The total score ranged from 0 to 100. Final scores were categorized as: > 80 (“healthy”), 50–80 (“needs improvement”), and <50 (“unhealthy”). Nevertheless, these cut-off points should be interpreted cautiously, as they have not been specifically validated in Peruvian populations or among university students. The HEI has demonstrated a Cronbach’s alpha of 0.67 in U.S. populations [30] and is designed as a quantitative assessment of overall diet quality [31].

Other covariates that were collected included sex, age, marital status, nationality, academic year, current living arrangement, prior diagnosis of anxiety, prior diagnosis of depression, and current use of psychiatric medication.

### Procedures

Prior to data collection, the study protocol was approved by the university’s ethics committee. Data collection was conducted from 15/07/2025to 15/12/2025, corresponding to the second academic semester at Universidad Peruana Unión. This period includes regular academic activities and the end-of-semester examination phase, during which students are often exposed to constant evaluations and sustained academic stress, both of which may influence dietary behaviors and mental health symptom burden. The survey, created using Google Forms, was distributed through academic year representatives. Additionally, in-person administration was conducted during academic hours to increase coverage and response rates. Participation was encouraged through a raffle of monetary incentives. Before completing the questionnaire, participants were informed about the study objectives and provided written informed consent. The structured questionnaire included sections on informed consent, sociodemographic data, dietary habits assessed with the HEI, and the PHQ-9 and GAD-7 scales.

### Statistical analysis

Data were cleaned in Microsoft Excel and subsequently analyzed using Stata version 19.0. Categorical variables were presented as absolute and relative frequencies, whereas numerical variables were presented as mean and standard deviation or median and interquartile range, depending on distribution. For bivariate analyses according to the presence of depression or anxiety, chi-square or Fisher’s exact tests were used for categorical variables, and Student’s t-test or the Mann–Whitney U test for continuous variables, as appropriate. The association between the Healthy Eating Index score and the prevalence of depression or anxiety was evaluated using Poisson regression with robust variance, estimating crude (cPR) and adjusted prevalence ratios (aPR) with 95% confidence intervals (95% CI). Before fitting the regression models, the linearity assumption between the HEI score and each outcome was assessed using the “lincheck” command. The categorization of the HEI score as unhealthy, needs improvement, and healthy was applied only for descriptive analyses. As a sensitivity analysis, the same adjusted Poisson regression models were refitted using 99% confidence intervals (99% CI) in order to evaluate the robustness of the estimated associations under a more conservative precision threshold. Variables considered potential confounders based on theoretical and epidemiological criteria were included in the adjusted model [32–34]. Marital status was not included in the adjusted model because it had very few observations in some categories, and most participants were single. Multicollinearity among the independent variables was formally assessed using the variance inflation factor (VIF). When age was initially modelled as a continuous variable, a VIF of 25.7 was observed, indicating severe collinearity; therefore, age was recategorized into tertiles for inclusion in the adjusted analyses, after which all VIF values were below 10. Post-estimation graphs were generated to assess the relationship between the HEI score and the prevalence of depression and anxiety. A p-value <0.05 was considered statistically significant.

### Ethical considerations

The study was conducted in accordance with the ethical principles for research involving human subjects established in the Declaration of Helsinki. The protocol was reviewed and approved by the Research Ethics Committee of the Faculty of Health Sciences at Universidad Peruana Unión (approval code: 2024-CEB-FCS - UPeU-«N°178»), and institutional authorization was obtained. Participation was voluntary, and written informed consent was obtained from all participants. Survey responses and data were collected anonymously and maintained confidentially.

## Results

A total of 264 medical students were surveyed, with no exclusions due to missing data. The median age was 21 years, with the majority being female (62.5%), single (96.6%), of Peruvian nationality (93.9%), and in the first year of study (23.5%). Regarding prior history, 6.8% reported a previous diagnosis of anxiety, 9.8% of depression, and 6.4% reported current use of psychiatric medication at the time of the survey. With respect to dietary habits, most students needed to make changes to their diet (77.3%), and a minority had healthy dietary habits (1.1%) (Table 1). The frequencies of food consumption by item are detailed in S1 Table.

**Table 1:** Characteristics of medical students at a private university in Lima, Peru (n = 264).

Characteristics	n (%)
Sex	
Male	99 (37.5)
Female	165 (62.5)
Age (years), median [IQR]	21 [19 to 23]
Marital status	
Single	255 (96.6)
Married	5 (1.9)
Cohabiting	4 (1.5)
Nationality	
Peru	248 (93.9)
Non-Peruvian	16 (6.1)
Academic year	
First year	62 (23.5)
Second year	57 (21.6)
Third year	31 (11.7)
Fourth year	32 (12.1)
Fifth year	40 (15.2)
Sixth year	42 (15.9)
Current living arrangement	
Alone	128 (48.5)
Nuclear family	107 (40.5)
Extended family	8 (3.0)
Friends/partner	21 (8.0)
Previous diagnosis of anxiety	
No	246 (93.2)
Yes	18 (6.8)
Previous diagnosis of depression	
No	238 (90.2)
Yes	26 (9.8)
Current psychiatric medication use	
No	247 (93.6)
Yes	17 (6.4)
Total dietary habits score, mean ± SD	56.9 ± 11.0
Dietary habits	
Unhealthy	57 (21.6)
Needs improvement	204 (77.3)
Healthy	3 (1.1)
Anxiety (GAD-7 ≥ 10)	
No	172 (65.2)
Yes	92 (34.9)
Depression (PHQ-9 ≥ 10)	
No	145 (54.9)
Yes	119 (45.1)

IQR: interquartile range; SD: standard deviation; GAD-7: Generalized Anxiety Disorder-7; PHQ-9: Patient Health Questionnaire-9.

The prevalence of anxiety was 34.9%, being more frequent among those with unhealthy dietary habits (63.2%, p < 0.001) and lower scores on the healthy eating scale (mean 52.2 ± 10.1, p < 0.001). In addition, it was significantly more prevalent among younger students (median 20: IQR [19 to 23], p = 0.042), those in basic sciences years (42.9%, p = 0.013), and those currently using psychiatric medication (76.5%, p < 0.001) (Table 2). The frequencies of anxiety symptoms are detailed in S2 Table.

**Table 2:** Bivariate analysis between sample characteristics and anxiety (n = 264).

Characteristics	Anxiety		p-value*
	Absent 172 (65.2%) n (%)	Present 92 (34.9%) n (%)	
Sex			0.894
Male	65 (65.7)	34 (34.3)	
Female	107 (64.9)	58 (35.1)	
Age (years), median [IQR]	21 (19–24)	20 (19–23)	0.042
Academic year			0.013
Basic sciences (first to second year)	68 (57.1)	51 (42.9)	
Clinical sciences (third to sixth year)	104 (71.7)	41 (28.3)	
Current living arrangement			0.288
Alone	78 (60.9)	50 (39.1)	
Nuclear/extended family	78 (67.8)	37 (32.2)	
Friends/partner	16 (76.2)	5 (23.8)	
Previous diagnosis of anxiety			0.056
No	164 (66.7)	82 (33.3)	
Yes	8 (44.4)	10 (55.6)	
Previous diagnosis of depression			0.401
No	157 (66.0)	81 (34.0)	
Yes	15 (57.7)	11 (42.3)	
Current psychiatric medication use			<0.001
No	168 (68.0)	79 (32.0)	
Yes	4 (23.5)	13 (76.5)	
Total dietary habits score, mean ± SD	59.4 ± 10.7	52.2 ± 10.1	<0.001
Dietary habits			<0.001
Unhealthy	21 (36.8)	36 (63.2)	
Needs improvement	148 (72.6)	56 (27.5)	
Healthy	3 (100)	0 (0)	

SD: standard deviation; IQR: interquartile range.

*p-value calculated using chi-square test, Fisher’s exact test, Student’s t-test, or Mann–Whitney U test.

Depression had a prevalence of 45.1%, being more frequent among students with unhealthy dietary patterns (71.9%, p < 0.001) and among those with lower scores on the healthy eating scale (mean 53.7 ± 11.1, p < 0.001). Likewise, a higher prevalence was observed among those in basic sciences years (54.6%, p = 0.005) and among students who reported current use of psychiatric medication (70.6%, p = 0.029) (Table 3). The frequencies of depressive symptoms are detailed in S3 Table.

**Table 3:** Bivariate analysis between sample characteristics and depression (n = 264).

Characteristics	Depression		p-value*
	Absent 145 (54.9%) n (%)	Present 119 (45.1%) n (%)	
Sex			0.389
Male	51 (51.5)	48 (48.5)	
Female	94 (57.0)	71 (43.0)	
Age (years), median [IQR]	21 (19–23)	20 (19–23)	0.098
Academic year			0.005
Basic sciences (first to second year)	54 (45.4)	65 (54.6)	
Clinical sciences (third to sixth year)	91 (62.8)	54 (37.2)	
Current living arrangement			0.230
Alone	64 (50.0)	64 (50.0)	
Nuclear/extended family	67 (58.3)	48 (41.7)	
Friends/partner	14 (66.7)	7 (33.3)	
Previous diagnosis of anxiety			0.157
No	138 (56.1)	108 (43.9)	
Yes	7 (38.9)	11 (61.1)	
Previous diagnosis of depression			0.076
No	135 (56.7)	103 (43.3)	
Yes	10 (38.5)	16 (61.5)	
Current psychiatric medication use			0.029
No	140 (56.7)	107 (43.3)	
Yes	5 (29.4)	12 (70.6)	
Total dietary habits score, mean ± SD	59.6 ± 10.2	53.7 ± 11.1	<0.001
Dietary habits			<0.001
Unhealthy	16 (28.1)	41 (71.9)	
Needs improvement	127 (62.3)	77 (37.8)	
Healthy	2 (66.7)	1 (33.3)	

SD: standard deviation; IQR: interquartile range.

*p-value calculated using chi-square test, Fisher’s exact test, Student’s t-test, or Mann–Whitney U test.

**Fig 1:**
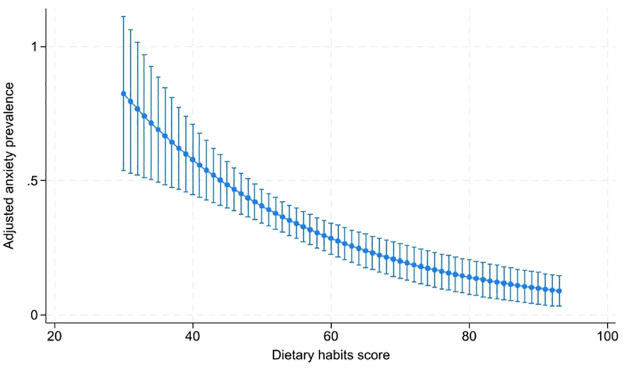
Relationship between healthy eating score and adjusted prevalence of anxiety.

**Fig 2:**
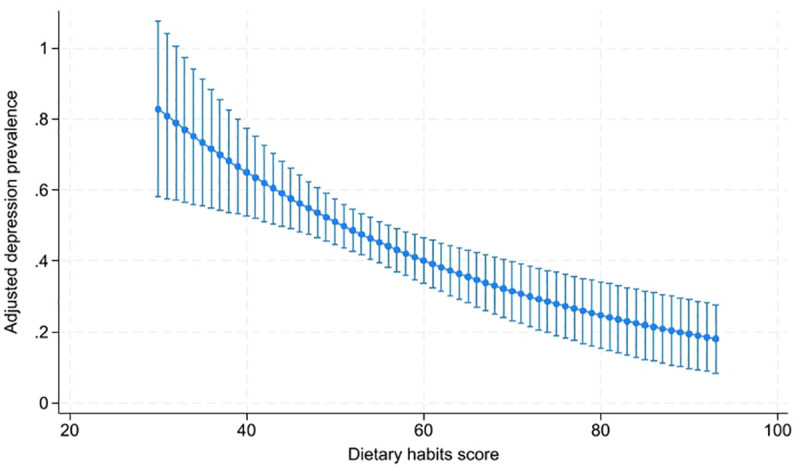
Relationship between healthy eating score and adjusted prevalence of depression.

In the adjusted regression analysis, healthier dietary habits were associated with a lower prevalence of both anxiety and depression. Specifically, for each additional point on the HEI scale, the prevalence of anxiety decreased by 3% (aPR: 0.97; 95% CI: 0.95 to 0.98; p < 0.001), while the prevalence of depression decreased by 2% (aPR: 0.98; 95% CI: 0.96 to 0.99; p < 0.001) (Table 4). In the sensitivity analysis using 99% CIs, the estimates remained highly statistically significant (anxiety: aPR: 0.97; 99% CI: 0.95 to 0.98; p < 0.001; depression: aPR: 0.98; 99% CI: 0.96 to 0.99; p < 0.001). Likewise, the graphical representation shows an inversely proportional relationship between healthy dietary habit scores and the prevalence of anxiety and depression (Figs 1 and 2).

**Table 4:** Association between healthy dietary habits score and the prevalence of depression and anxiety (n = 264).

Variable	Crude PR (IC 95%)	p-value	Adjusted PR (IC 95%)*	p-value
Anxiety prevalence	0.96 (0.95 to 0.98)	<0.001	0.97 (0.95 to 0.98)	<0.001
Depression prevalence	0.97 (0.96 to 0.99)	<0.001	0.98 (0.96 to 0.99)	<0.001

PR: prevalence ratio; 95% CI: 95% confidence interval.

*Adjusted for age, sex, academic year, current living arrangement, previous diagnosis of anxiety, previous diagnosis of depression, and current use of psychiatric medication.

## Discussion

### Main findings

In the present study conducted among Peruvian medical students, a high prevalence of depressive and anxiety symptoms was observed, as well as a high frequency of dietary habits that did not meet nutritional recommendations. Additionally, healthier dietary habits were inversely associated with the prevalence of anxiety and depression.

Similarly, a systematic review of university students that included more than 100 000 participants reported a prevalence of depressive symptoms of 33.6% and anxiety symptoms of 39.0%, with higher prevalences observed among medical students and in low- and middle-income countries [35]. Other studies conducted among students in Peru have reported even higher prevalences, exceeding 60% for these disorders [36,37]. These elevated figures may be explained by high academic demands, adaptation difficulties, lack of physical activity, family problems, financial constraints, social instability, and limited access to specialized mental health services [38,39]. Additionally, it is noteworthy that, unlike most previous reports, depression prevalence in our study was higher than anxiety prevalence. One possible explanation is that prolonged exposure to chronic academic stress may promote a state of emotional exhaustion more closely aligned with depressive symptomatology than with acute anxiety [40].

The low proportion of students classified as having healthy dietary habits is consistent with previous Latin American evidence. In Peru, a study from Huancavelica using the HEI found that only 0.8% of adults had a healthy diet [41]. Similarly, among Chilean university students, only 9.3% had a healthy diet, while 55.3% needed changes and 35.4% had an unhealthy diet [42]. In Mexico, only 1% of adolescent women were classified as having good diet quality using an adapted HEI [43]. This may reflect the difficulty of meeting strict healthy eating thresholds in real-world student populations, as well as irregular eating schedules, academic stress, limited time for meal planning, and high availability of ultra-processed foods and sugar-sweetened beverages.

Regarding the association between dietary habits and anxiety, a systematic review with a search date up to 2022 included 45 studies, of which 36 reported that better diet quality was associated with lower levels of depression, anxiety, and stress. However, stress and anxiety were also associated with poorer dietary habits, suggesting a bidirectional relationship [20]. Similarly, another systematic review conducted among adolescents found consistent results linking better diet quality with improved mental health outcomes [44]. In our study, this association was also supported by the strength and consistency of the findings, as each additional point in the HEI was associated with a 3% lower prevalence of anxiety, and this association remained highly statistically significant in the sensitivity analysis using 99% CI. Additionally, evidence from several systematic reviews indicates that the consumption of processed foods or fast food is associated with worse mental health outcomes [45,46]. In this context, plausible biological pathways proposed in the literature suggest that healthy dietary patterns may protect against depression and anxiety by reducing inflammation and oxidative stress, improving metabolic health, and modulating the gut–brain axis and neurotransmitter regulation [47,48]. On the other hand, high consumption of ultra-processed foods may contribute to worsening mental health by promoting inflammation, dysregulation of the hypothalamic–pituitary–adrenal axis, and alterations in the synthesis of serotonin, dopamine, and norepinephrine [14].

### Implications and recommendations

Our findings may be generalizable to similar university settings where medical students facing high academic demands exhibit a high prevalence of depressive (45.1%) and anxiety (34.9%) symptoms, along with a high frequency of dietary patterns that “need improvement” (77.3%) or are considered “unhealthy” (21.6%). However, the study context should be considered when interpreting external validity. This university is sponsored by the Seventh-day Adventist Church, which promotes a predominantly plant-based diet, regular physical activity, and abstinence from alcohol and tobacco. As a result, students may be influenced by institutional, religious, and peer-related factors that shape dietary habits differently from those universities without a religious affiliation. Notably, these disorders were more frequent during the early years of study, suggesting that this subgroup may represent a particularly vulnerable population. Our findings, together with the existing literature, suggest that diet quality may be a relevant factor to consider in the development of comprehensive student well-being strategies, including periodic mental health screening, nutritional counselling and education, and improved availability of healthy food in university cafeterias. Finally, multicenter longitudinal studies are needed to confirm the directionality of this association, and ideally, clinical trials should evaluate the impact of healthy dietary programs on mental health outcomes.

### Limitations and strengths

Our study used a non-probabilistic convenience sampling approach, which limits the generalizability of the findings. Furthermore, as this was a single-center study conducted at an Adventist institution, the extrapolation of the results to other settings may be limited. The cross-sectional design precludes establishing temporality and directionality between dietary habits and symptoms of anxiety and depression, hence reverse causality is plausible, as symptoms of depression and anxiety are known to alter appetite, food preferences, and eating behavior, which may result in poorer diet quality. Although validated instruments were used, the PHQ-9 and GAD-7 do not replace a clinical reference diagnosis. Regarding dietary exposure, the Spanish-adapted HEI has not been culturally validated in Peru or among Peruvian university students, which may have introduced non-differential exposure misclassification because it may not fully capture local dietary patterns, portion sizes, cooking methods, sodium intake, or degree of food processing. Also, its categorical cut-offs have not been standardized for the Peruvian population, which may explain the very low proportion of students classified as having ‘healthy’ dietary habits (1.1%), although this finding is consistent with previous Latin American evidence. In addition, residual confounding is an important limitation. Variables associated with both dietary habits and mental health in university students, such as sleep quality, physical activity, academic stress, and socioeconomic status, were not measured and therefore could not be included in the adjusted models. This is particularly relevant because data collection included the end-of-semester examination phase, when academic stress, sleep disruption, reduced physical activity, limited time for meal preparation, and worsening mental health symptoms may co-occur. Therefore, unmeasured academic and socioeconomic factors may partly explain the observed association between diet quality and anxiety or depression, potentially leading to underestimation or overestimation of the observed associations. On the other hand, we adjusted for the main potential confounding variables, thereby strengthening the internal validity of the findings. In addition, the strength of the association, even with 99% CI, and the inversely proportional relationship supports a potential association between the variables. Finally, this study addressed a population that has been underexplored in the Peruvian context, where the magnitude of the association may be particularly relevant.

## Conclusion

Among Peruvian medical students, a high prevalence of depressive and anxiety symptoms was observed, along with a high frequency of dietary habits that did not meet nutritional recommendations. Healthier dietary habits were inversely associated with the prevalence of depression and anxiety. These findings suggest that promoting healthy dietary patterns should be considered as part of comprehensive strategies aimed at improving mental health among medical students.

## Supporting information

S1 Table Frequency of food consumption according to the healthy eating index. (DOCX)

S2 Table Frequency of anxiety symptoms according to GAD-7 items (n = 264). (DOCX)

S3 Table Frequency of depressive symptoms according to PHQ-9 items (n = 264). (DOCX)

S1 File Data. (XLSX)
