# Supplementary material for: Healthier dietary habits are associated with lower depression and anxiety among medical students at a private university in Lima, Peru: A cross-sectional study
Source: PLoS One. 2026 Jun 18;21(6):e0346062. doi: 10.1371/journal.pone.0346062 (PMC13278413; doi:10.1371/journal.pone.0346062)
Supplement: S1 Table — (DOCX) [file pone.0346062.s001.docx]

S1 Table. Frequency of food consumption according to the healthy eating index.

| Daily consumption (recommended foods) | | 10 pts (Daily) n (%) | 7.5 pts (≥3 times/week, not daily) n (%) | 5 pts (1–2 times/week) n (%) | 5 pts (1–2 times/week) n (%) | 5 pts (1–2 times/week) n (%) |
| --- | --- | --- | --- | --- | --- | --- |
|  | Cereals and grains | 49 (18.6) | 89 (33.7) | 83 (31.4) | 30 (11.4) | 13 (4.9) |
|  | Vegetables and greens | 76 (28.8) | 116 (43.9) | 60 (22.7) | 10 (3.8) | 2 (0.8) |
|  | Fruits | 55 (20.8) | 85 (32.2) | 92 (34.9) | 28 (10.6) | 4 (1.5) |
|  | Milk and dairy products | 30 (11.4) | 73 (27.7) | 99 (37.5) | 49 (18.6) | 13 (4.9) |
|  |  |  |  |  |  |  |
| Weekly consumption | | 10 pts (1–2 times/week) n (%) | 7.5 pts (≥3 times/week, not daily) n (%) | 5 pts (<1 time/week) n (%) | 2.5 pts (Daily) n (%) | 0 pts (Never/almost never) n (%) |
|  | Meat | 36 (13.6) | 110 (41.7) | 54 (20.5) | 38 (14.4) | 26 (9.8) |
|  | Legumes | 53 (20.1) | 135 (51.1) | 47 (17.8) | 29 (11.0) | 0 (0) |
|  |  |  |  |  |  |  |
| Occasional consumption (non-recommended foods) | | 10 pts (Never/almost never) n (%) | 7.5 pts (<1 time/week) n (%) | 5 pts (1–2 times/week) n (%) | 2.5 pts (≥3 times/week, not daily) n (%) | 0 pts (Daily) n (%) |
|  | Processed meats and cold cuts | 73 (27.7) | 88 (33.3) | 67 (25.4) | 32 (12.1) | 4 (1.5) |
|  | Sweets | 31 (11.7) | 88 (33.3) | 86 (32.6) | 47 (17.8) | 12 (4.6) |
|  | Sugar-sweetened beverages | 40 (15.2) | 77 (29.2) | 77 (29.2) | 51 (19.3) | 19 (7.2) |
