# Supplementary material for: Healthier dietary habits are associated with lower depression and anxiety among medical students at a private university in Lima, Peru: A cross-sectional study
Source: PLoS One. 2026 Jun 18;21(6):e0346062. doi: 10.1371/journal.pone.0346062 (PMC13278413; doi:10.1371/journal.pone.0346062)
Supplement: S2 Table — (DOCX) [file pone.0346062.s002.docx]

S2 Table. Frequency of anxiety symptoms according to GAD-7 items (n = 264).

| GAD-7 Item | Not at all  n (%) | Less than half the days  n (%) | More than half the days  n (%) | Nearly every day  n (%) |
| --- | --- | --- | --- | --- |
| GAD-1. Feeling nervous, anxious, or on edge | 50 (18.9) | 122 (46.2) | 82 (31.1) | 10 (3.8) |
| GAD-2. Not being able to stop or control worrying | 63 (23.9) | 122 (46.2) | 70 (26.5) | 9 (3.4) |
| GAD-3. Worrying too much about different things | 54 (20.5) | 110 (41.7) | 86 (32.6) | 14 (5.3) |
| GAD-4. Trouble relaxing | 54 (20.5) | 126 (47.7) | 72 (27.3) | 12 (4.6) |
| GAD-5. Being so restless that it is hard to sit still | 83 (31.4) | 117 (44.3) | 55 (20.8) | 9 (3.4) |
| GAD-6. Becoming easily annoyed or irritable | 53 (20.1) | 123 (46.6) | 76 (28.8) | 12 (4.6) |
| GAD-7. Feeling afraid as if something awful might happen | 77 (29.2) | 110 (41.7) | 63 (23.9) | 14 (5.3) |
