# Supplementary material for: Healthier dietary habits are associated with lower depression and anxiety among medical students at a private university in Lima, Peru: A cross-sectional study
Source: PLoS One. 2026 Jun 18;21(6):e0346062. doi: 10.1371/journal.pone.0346062 (PMC13278413; doi:10.1371/journal.pone.0346062)
Supplement: S3 Table — (DOCX) [file pone.0346062.s003.docx]

S3 Table. Frequency of depressive symptoms according to PHQ-9 items (n = 264).

| PHQ-9 Item | Not at all  n (%) | Less than half the days  n (%) | More than half the days  n (%) | Nearly every day  n (%) |
| --- | --- | --- | --- | --- |
| PHQ-1. Little interest or pleasure in doing things | 76 (28.8) | 100 (37.9) | 72 (27.3) | 16 (6.1) |
| PHQ-2. Feeling down, depressed, or hopeless | 93 (35.2) | 103 (39.0) | 57 (21.6) | 11 (4.2) |
| PHQ-3. Sleep disturbances | 91 (34.5) | 88 (33.3) | 70 (26.5) | 15 (5.7) |
| PHQ-4. Feeling tired or having little energy | 44 (16.7) | 113 (42.8) | 79 (29.9) | 28 (10.6) |
| PHQ-5. Appetite disturbances | 69 (26.1) | 100 (37.9) | 74 (28.0) | 21 (8.0) |
| PHQ-6. Feeling bad about yourself | 82 (31.1) | 94 (35.6) | 67 (25.4) | 21 (8.0) |
| PHQ-7. Trouble concentrating | 75 (28.4) | 95 (36.0) | 78 (29.6) | 16 (6.1) |
| PHQ-8. Psychomotor agitation or retardation | 129 (48.9) | 74 (28.0) | 49 (18.6) | 12 (4.6) |
| PHQ-9. Thoughts of death or self-harm | 153 (58.0) | 59 (22.4) | 40 (15.2) | 12 (4.6) |
