# Supplementary material for: Healthier dietary habits are associated with lower depression and anxiety among medical students at a private university in Lima, Peru: A cross-sectional study
Source: PLoS One. 2026 Jun 18;21(6):e0346062. doi: 10.1371/journal.pone.0346062 (PMC13278413; doi:10.1371/journal.pone.0346062)
Supplement: Attachment — Submitted filename: Response to reviewers.docx [file pone.0346062.s005.docx]

**RESPONSE TO REVIEWERS**

**Manuscript title:** Healthier dietary habits are associated with lower depression and anxiety among medical students at a private university in Lima, Peru: A cross-sectional study

**Manuscript ID:** PONE-D-26-09038

We sincerely thank the Academic Editor, and both reviewers for their thorough, rigorous, and constructive evaluation of our manuscript. Their comments identified areas requiring greater methodological transparency, more cautious interpretation of findings, and more explicit discussion of the study’s limitations. We have addressed each concern systematically in the response below, and we believe the revised manuscript is substantially stronger because of this process.

**ACADEMIC EDITOR COMMENTS**

**Comment AE 1.1.** “Clarify and justify the analytical approach. Provide a clear description of the variable selection strategy for regression models (e.g., theoretical criteria, epidemiological relevance, or statistical thresholds). Explain how multicollinearity was assessed and handled—the manuscript mentions categorizing age into tertiles due to multicollinearity, but the diagnostics are not described. Report goodness-of-fit measures appropriate for Poisson regression with robust variance.”

**Response:** We appreciate this important observation. Covariates for the adjusted regression models were selected a priori on the basis of theoretical association with the outcomes and epidemiological relevance, rather than on statistical cut-offs such as p-values in the bivariate analysis. We recognize that this strategy was not sufficiently described in the original manuscript and have expanded the Statistical analysis section accordingly, adding references supporting the selection of each covariate. Multicollinearity among the independent variables included in each adjusted model was formally evaluated using the variance inflation factor (VIF). When age was initially entered as a continuous variable, a VIF of 25.7 was observed, indicating severe multicollinearity. To address this, age was recategorized into tertiles and included in this categorical form in the adjusted models, after which all VIF values were below the conventional threshold of 10. Regarding goodness-of-fit, we respectfully note that the aim of our analysis was etiologic/associational rather than predictive. Consequently, the regression models were not intended to maximize predictive performance but to estimate the association between dietary habits and the prevalence of anxiety and depression while adjusting for theoretically relevant confounders. For this reason, goodness-of-fit indices were not a primary focus of the analysis. We have clarified this analytical purpose in the revised Methods.

**Modified text in manuscript (Methods – Statistical analysis):**

“The association between the Healthy Eating Index score and the prevalence of depression or anxiety was evaluated using Poisson regression with robust variance, estimating crude (cPR) and adjusted prevalence ratios (aPR) with 95% confidence intervals (95% CI). Variables considered potential confounders based on theoretical and epidemiological criteria were included in the adjusted model [31–33]. Marital status was not included in the adjusted model because it had very few observations in some categories, and most participants were single. Multicollinearity among the independent variables was formally assessed using the variance inflation factor (VIF). When age was initially modelled as a continuous variable, a VIF of 25.7 was observed, indicating severe collinearity; therefore, age was recategorized into tertiles for inclusion in the adjusted analyses, after which all VIF values were below 10.”

**Comment AE 1.2.** “Address concerns regarding the dietary assessment instrument. Only 1.1% of participants were classified as having ‘healthy’ dietary habits. This extremely low proportion suggests potential misclassification or lack of calibration of the Spanish-adapted HEI for this population. Provide a stronger justification for using the Spanish-adapted HEI; discuss exposure misclassification including how cultural and dietary differences may affect scoring; consider a sensitivity analysis using tertiles or quartiles of HEI score.”

**Response:** We fully agree with the Academic Editor that the Spanish-adapted HEI provides categorical cut-offs that have not been standardized or formally calibrated for the Peruvian population, and this represents an important limitation of the instrument. For this reason, the three categories (unhealthy, needs improvement, healthy) were reported descriptively only, to characterize the sample and to enable comparison with previous literature. Crucially, the regression models estimating the association between dietary habits and anxiety/depression were not based on these categories but on the HEI total score modelled as a continuous variable. This analytical choice avoids reliance on cut-offs that have not been validated in Peruvian youth and preserves the full informational content of the scale. For the same reason, we respectfully consider that a complementary analysis using tertiles or quartiles of the HEI score would not add substantive information beyond what the linear model and its post-estimation plots already convey and could in fact reintroduce arbitrary categorizations of the exposure. We selected the Spanish-adapted HEI because it is the best-documented instrument available in Spanish for quantitative assessment of overall diet quality, and because the dietary recommendations underlying its scoring are closer to the Peruvian dietary context than those of purely Anglo-American instruments. We have added a clear acknowledgement of exposure misclassification and of the lack of Peruvian calibration of the categorical cut-offs to the Methods and the Limitations section.

**Modified text in manuscript (Methods – Variables):**

“The total score ranged from 0 to 100. Final scores were categorized as: >80 (“healthy”), 50–80 (“needs improvement”), and <50 (“unhealthy”). Nevertheless, these cut-off points should be interpreted cautiously, as they have not been specifically validated in Peruvian populations or among university students.”

**Modified text in manuscript (Limitations and strengths):**

“Also, the categorical cut-offs proposed for the Spanish-adapted HEI have not been standardized for the Peruvian population. This may help explain the very low proportion of students classified as having ‘healthy’ dietary habits (1.1%).”

**Comment AE 1.3.** “Revise interpretation to avoid causal implications. The manuscript suggests a ‘dose–response effect’ and discusses biological mechanisms in a way that implies causality. Remove or rephrase language implying temporal ordering or causal inference; expand the discussion of reverse causation; ensure biological mechanisms are presented as hypothetical.”

**Response:** We agree and have carefully revised the language throughout the manuscript. The term “dose–response effect” has been removed and replaced, where appropriate, with “inverse linear relationship” or “inversely proportional relationship” between the healthy eating score and the prevalence of anxiety and depression, which better reflects the statistical behaviour of the association. Statements that could imply temporal ordering or causal inference have been softened or removed. Biological mechanisms are now explicitly framed as hypothetical explanations supported by previous literature rather than as findings of the present study. Furthermore, we have expanded the discussion of reverse causation, acknowledging that symptoms of anxiety and depression may themselves modify dietary behaviour.

**Modified text in manuscript (Limitations and strengths):**

“Second, the cross-sectional design precludes establishing temporality and directionality between dietary habits and symptoms of anxiety and depression, hence reverse causality is plausible, as symptoms of depression and anxiety are known to alter appetite, food preferences, and eating behavior, which may result in poorer diet quality.”

“In addition, the strength of the association and the inversely proportional relationship supports a potential association between the variables.”

**Modified text in manuscript (Discussion – second paragraph on mechanisms):**

“In this context, plausible biological pathways proposed in the literature suggest that healthy dietary patterns may protect against depression and anxiety by reducing inflammation and oxidative stress, improving metabolic health, and modulating the gut–brain axis and neurotransmitter regulation [43,44]. On the other hand, high consumption of ultra-processed foods may contribute to worsening mental health by promoting inflammation, dysregulation of the hypothalamic–pituitary–adrenal axis, and alterations in the synthesis of serotonin, dopamine, and norepinephrine [14].”

**Comment AE 1.4.** “Discuss unmeasured confounding. The adjusted models do not include several important confounders (sleep quality, physical activity, academic stress, socioeconomic status, BMI). Provide a clear justification for their absence and expand the discussion of residual confounding.”

**Response:** We thank the Academic Editor for this well-taken observation. We acknowledge that sleep quality, physical activity, academic stress, and socioeconomic status were not collected in this study and therefore could not be included in the adjusted models, which is a genuine limitation that we have added explicitly to the Limitations section. We also note that residual confounding by the variables mentioned by the Academic Editor may have led to either underestimation or overestimation of the observed association. As is often the case, adjustment for additional correlated lifestyle confounders (e.g., physical activity, sleep) might move the effect estimate somewhat toward the null, though the direction and magnitude cannot be anticipated with certainty. An important distinction that we would like to raise is that body mass index (BMI) is most plausibly a mediator rather than a confounder of the relationship between dietary habits and mental health, since diet quality is an established determinant of BMI, and BMI itself has been linked to depressive and anxiety symptoms. Adjusting for BMI could therefore produce overadjustment bias and partially block the total effect of diet quality on mental health. For these reasons, BMI would not have been included in the adjusted model even if it had been available. These considerations have been added to the revised Discussion.

**Modified text in manuscript (Limitations and strengths):**

“In addition, residual confounding is an important limitation. Variables associated with both dietary habits and mental health in university students, such as sleep quality, physical activity, academic stress, and socioeconomic status, were not measured and therefore could not be included in the adjusted models. This may have led to underestimation or overestimation of the observed associations.”

**Comment AE 1.5.** “Emphasize effect sizes and confidence intervals. Highlight prevalence ratios and confidence intervals as the primary indicators of association; avoid interpreting results solely based on statistical significance.”

**Response:** We fully agree. The Results and Discussion have been revised to place the adjusted prevalence ratios and their 95% confidence intervals at the center of interpretation, rather than focusing on p-values alone. To further reinforce the robustness and precision of the estimates, we added a sensitivity analysis using 99% confidence intervals. The results remained consistent: anxiety aPR = 0.97 (99% CI: 0.95 to 0.98; p<0.001) and depression aPR = 0.98 (99% CI: 0.96 to 0.99; p<0.001). We have added this sensitivity analysis to the Methods, Results, and Discussion sections, and we have reframed the interpretation of the findings around effect size and precision. We wish to note, however, that we have been careful not to overclaim certainty or causality on the basis of these findings.

**Modified text in manuscript (Methods – Statistical analysis):**

“As a sensitivity analysis, the same adjusted Poisson regression models were refitted using 99% confidence intervals (99% CI) in order to evaluate the robustness of the estimated associations under a more conservative precision threshold.”

**Modified text in manuscript (Results):**

“In the sensitivity analysis using 99% CIs, the estimates remained highly statistically significant (anxiety: aPR: 0.97; 99% CI: 0.95 to 0.98; p<0.001; depression: aPR: 0.98; 99% CI: 0.96 to 0.99; p<0.001).”

**Modified text in manuscript (Discussion – Main findings):**

“In our study, this association was also supported by the strength and consistency of the findings, as each additional point in the HEI was associated with a 3% lower prevalence of anxiety, and this association remained highly statistically significant in the sensitivity analysis using 99% CI.”

**Comment AE 2.1.** “[Recommended] Expand the discussion of generalizability, considering the single-center, convenience sample and the unique characteristics of a Seventh-day Adventist institution.”

**Response:** We agree and have expanded the discussion of external validity in the Limitations and in the Implications and recommendations sections. The study was conducted at a single institution, with non-probabilistic convenience sampling, in medical students from a university sponsored by the Seventh-day Adventist Church. The Adventist tradition explicitly promotes a predominantly plant-based dietary pattern, regular physical activity, and abstinence from alcohol and tobacco as part of its health message.

**Modified text in manuscript (Implications and recommendations):**

“Our findings may be generalizable to similar university settings where medical students facing high academic demands exhibit a high prevalence of depressive (45.1%) and anxiety (34.9%) symptoms, along with a high frequency of dietary patterns that “need improvement” (77.3%) or are considered “unhealthy” (21.6%). However, the study context should be considered when interpreting external validity. This university is sponsored by the Seventh-day Adventist Church, which promotes a predominantly plant-based diet, regular physical activity, and abstinence from alcohol and tobacco. As a result, students may be influenced by institutional, religious, and peer-related factors that shape dietary habits differently from those in secular universities.”

**Comment AE 3.1.** “[Editorial Note] Clarify the data collection period in relation to the academic calendar, as mental health symptoms may vary across semesters.”

**Response:** We agree and have clarified the data collection period and its position within the academic calendar in the Methods section. We have also noted that the window included the end-of-semester examination phase, which is typically associated with heightened academic stress and may influence both dietary behavior and mental health symptom burden.

**Modified text in manuscript (Methods – Procedures):**

“Data collection was conducted from 15/07/2025 to 15/12/2025, corresponding to the second academic semester at Universidad Peruana Unión. This period includes regular academic activities and the end-of-semester examination phase, during which students are often exposed to constant evaluations and sustained academic stress, both of which may influence dietary behaviors and mental health symptom burden.”

**Comment AE 3.2.** “[Editorial Note] Ensure consistency in reporting (e.g., whether HEI is treated as continuous, categorical, or both).”

**Response:** We thank the Academic Editor for this helpful observation. We have revised the manuscript to ensure consistency throughout. Specifically, we now state explicitly that: the HEI was used categorically for descriptive purposes only.

**Modified text in manuscript (Methods – Statistical analysis):**

“The categorization of the HEI score as unhealthy, needs improvement, and healthy was applied only for descriptive analyses.”

**REVIEWER #1 COMMENTS**

**Comment R1.1.** “The original dataset contains more than 30 variables, yet it is unclear how variables were selected for inclusion in the bivariate analyses and subsequent regression models. A systematic description of this variable selection process is needed.”

**Response:** We thank the Reviewer for highlighting this point. Most of the more than 30 variables in the original database correspond to the individual items of the PHQ-9 (9 items), GAD-7 (7 items), and the Spanish-adapted HEI (10 items), which together constitute the three composite instruments used to define the outcomes and the main exposure. The remaining variables correspond to sociodemographic and clinical characteristics (sex, age, marital status, academic year, current living arrangement, previous diagnosis of anxiety, previous diagnosis of depression, current psychiatric medication use) which were included in the bivariate and adjusted analyses primarily as potential confounders.

**Comment R1.2.** “The manuscript does not address how correlations among independent variables were handled in the regression analyses. If multicollinearity is present, the authors should describe the steps taken to assess and mitigate its impact.”

**Response:** We thank the Reviewer for this observation, which is consistent with the Academic Editor’s comment AE 1.1. As detailed in our response to that comment, covariates for the adjusted models were selected on a priori theoretical and epidemiological grounds. Multicollinearity among the independent variables was formally assessed using the variance inflation factor (VIF). When age was modelled as a continuous variable, a VIF of 25.7 was observed, indicating severe collinearity. To mitigate this, age was recategorized into tertiles and included in this form in the adjusted models; after recategorization, all VIF values were below the conventional threshold of 10. This procedure has been explicitly described in the revised Methods.

**Modified text in manuscript (Methods – Statistical analysis):**

“Multicollinearity among the independent variables was formally assessed using the variance inflation factor (VIF). When age was initially modelled as a continuous variable, a VIF of 25.7 was observed, indicating severe collinearity; therefore, age was recategorized into tertiles for inclusion in the adjusted analyses, after which all VIF values were below 10.”

**Comment R1.3.** “For all regression models, appropriate goodness-of-fit measures should be reported to help readers evaluate model adequacy.”

**Response:** We appreciate this observation. As noted in our response to AE 1.1, our aim was etiologic rather than predictive: the regression models were specified to estimate the association between dietary habits and the prevalence of anxiety and depression while adjusting for theoretically relevant confounders, not to maximize predictive performance or develop a prognostic model. For this reason, goodness-of-fit measures were not used to select or compare models.

**Comment R1.4.** “Reliance on p-values is not ideal in the context of exploratory analysis. Instead, the authors are encouraged to emphasize effect sizes along with 95% or 99% confidence intervals.”

**Response:** We fully agree, and this comment aligns with comment AE 1.5 from the Academic Editor. The Results and Discussion have been revised so that the adjusted prevalence ratios and their 95% confidence intervals are presented as the primary indicators of association, rather than relying on statistical significance. In addition, we performed the suggested sensitivity analysis re-estimating the main associations using 99% confidence intervals, with the following results: anxiety aPR = 0.97 (99% CI: 0.95 to 0.98; p<0.001) and depression aPR = 0.98 (99% CI: 0.96 to 0.99; p<0.001). The sensitivity analysis showed that the estimates remained similar under a more conservative confidence threshold. These results have been added to the Methods, Results, and Discussion. Importantly, in reframing the interpretation around effect sizes and confidence intervals, we have been cautious to avoid overclaiming certainty or implying causal inference.

**Modified text in manuscript (Methods – Statistical analysis):**

“As a sensitivity analysis, the same adjusted Poisson regression models were refitted using 99% confidence intervals (99% CI) in order to evaluate the robustness of the estimated associations under a more conservative precision threshold.”

**Modified text in manuscript (Results):**

“In the sensitivity analysis using 99% CIs, the estimates remained highly statistically significant (anxiety: aPR: 0.97; 99% CI: 0.95 to 0.98; p<0.001; depression: aPR: 0.98; 99% CI: 0.96 to 0.99; p<0.001).”

**Modified text in manuscript (Discussion – Main findings):**

“In our study, this association was also supported by the strength and consistency of the findings, as each additional point in the HEI was associated with a 3% lower prevalence of anxiety, and this association remained highly statistically significant in the sensitivity analysis using 99% CI.”

**REVIEWER #2 COMMENTS**

**Comment R2.1.** “The reliance on a single-centre, convenience sample from a private Adventist university severely limits external validity and introduces potential selection bias.”

**Response:** We agree with this concern, which is in line with comment AE 2.1. We have expanded the discussion of external validity and selection bias in the Limitations section. Specifically, we now explicitly acknowledge that the use of non-probabilistic convenience sampling in a single Seventh-day Adventist setting promotes a predominantly plant-based dietary pattern, regular physical activity, and abstinence from alcohol and tobacco, and students embedded in this environment may therefore display dietary profiles and mental health patterns that are not directly comparable to those of students in secular institutions.

**Modified text in manuscript (Implications and recommendations):**

“Our findings may be generalizable to similar university settings where medical students facing high academic demands exhibit a high prevalence of depressive (45.1%) and anxiety (34.9%) symptoms, along with a high frequency of dietary patterns that “need improvement” (77.3%) or are considered “unhealthy” (21.6%). However, the study context should be considered when interpreting external validity. This university is sponsored by the Seventh-day Adventist Church, which promotes a predominantly plant-based diet, regular physical activity, and abstinence from alcohol and tobacco. As a result, students may be influenced by institutional, religious, and peer-related factors that shape dietary habits differently from those universities without a religious affiliation.”

**Comment R2.2.** “Only 1.1% of participants were classified as having ‘healthy’ dietary habits, which suggests a lack of calibration of the instrument for this population. The use of a Spanish-adapted Healthy Eating Index may not be adequately justified for Peruvian medical students and may not reflect local dietary patterns.”

**Response:** We selected the Spanish-adapted HEI because it is the best-documented instrument available in Spanish for quantitative assessment of overall diet quality, and because the dietary recommendations underlying its scoring are closer to the Peruvian dietary context than those of purely Anglo-American instruments. We have added a clear acknowledgement of exposure misclassification and of the lack of Peruvian calibration of the categorical cut-offs to the Methods and the Limitations section.

**Modified text in manuscript (Methods – Variables):**

“The categorization of the HEI score as unhealthy, needs improvement, and healthy was applied only for descriptive analyses.”

**Modified text in manuscript (Limitations and strengths):**

“Also, the categorical cut-offs proposed for the Spanish-adapted HEI have not been standardized for the Peruvian population. This may help explain the very low proportion of students classified as having ‘healthy’ dietary habits (1.1%).”

**Comment R2.3.** “The authors invoke a ‘dose–response effect’ and suggest that the observed gradient supports a potential causal relationship, which is not justified given the cross-sectional design. Temporal ambiguity and reverse causation remain unresolved.”

**Response:** We agree and have carefully revised the language throughout the manuscript. The term “dose–response effect” has been removed and replaced, where appropriate, with “inverse linear relationship” or “inversely proportional relationship” between the healthy eating score and the prevalence of anxiety and depression, which better reflects the statistical behaviour of the association. Statements that could imply temporal ordering or causal inference have been softened or removed. Biological mechanisms are now explicitly framed as hypothetical explanations supported by previous literature rather than as findings of the present study. Furthermore, we have expanded the discussion of reverse causation, acknowledging that symptoms of anxiety and depression may themselves modify dietary behaviour.

**Modified text in manuscript (Limitations and strengths):**

“Second, the cross-sectional design precludes establishing temporality and directionality between dietary habits and symptoms of anxiety and depression, hence reverse causality is plausible, as symptoms of depression and anxiety are known to alter appetite, food preferences, and eating behavior, which may result in poorer diet quality.”

“In addition, the strength of the association and the inversely proportional relationship supports a potential association between the variables.”

**Modified text in manuscript (Discussion – second paragraph on mechanisms):**

“In this context, plausible biological pathways proposed in the literature suggest that healthy dietary patterns may protect against depression and anxiety by reducing inflammation and oxidative stress, improving metabolic health, and modulating the gut–brain axis and neurotransmitter regulation [43,44]. On the other hand, high consumption of ultra-processed foods may contribute to worsening mental health by promoting inflammation, dysregulation of the hypothalamic–pituitary–adrenal axis, and alterations in the synthesis of serotonin, dopamine, and norepinephrine [14].”

**Comment R2.4.** “The adjusted models omit several key confounders, including sleep, physical activity, academic stress, socioeconomic status, and BMI, increasing the likelihood of residual confounding.”

**Response:** We thank the reviewer for this well-taken observation. We acknowledge that sleep quality, physical activity, academic stress, and socioeconomic status were not collected in this study and therefore could not be included in the adjusted models, which is a genuine limitation that we have added explicitly to the Limitations section. We also note that residual confounding by the variables mentioned by the Academic Editor may have led to either underestimation or overestimation of the observed association. As is often the case, adjustment for additional correlated lifestyle confounders (e.g., physical activity, sleep) might move the effect estimate somewhat toward the null, though the direction and magnitude cannot be anticipated with certainty. An important distinction that we would like to raise is that body mass index (BMI) is most plausibly a mediator rather than a confounder of the relationship between dietary habits and mental health, since diet quality is an established determinant of BMI, and BMI itself has been linked to depressive and anxiety symptoms. Adjusting for BMI could therefore produce overadjustment bias and partially block the total effect of diet quality on mental health. For these reasons, BMI would not have been included in the adjusted model even if it had been available. These considerations have been added to the revised Discussion.

**Modified text in manuscript (Limitations and strengths):**

“In addition, residual confounding is an important limitation. Variables associated with both dietary habits and mental health in university students, such as sleep quality, physical activity, academic stress, and socioeconomic status, were not measured and therefore could not be included in the adjusted models. This may have led to underestimation or overestimation of the observed associations.”

**Comment R2.5.** “The discussion extends beyond the evidence by invoking biological mechanisms and policy implications that are not supported by the study design.”

**Response:** We appreciate this observation. We revised the manuscript to present both the biological mechanisms and the policy/practice implications more cautiously. Biological mechanisms are now framed as hypothetical explanations from prior literature, and the practical implications were softened to avoid prescriptive recommendations beyond the limits of the study.

**Modified text in manuscript (Discussion – mechanisms paragraph):**

“In this context, plausible biological pathways proposed in the literature suggest that healthy dietary patterns may protect against depression and anxiety by reducing inflammation and oxidative stress, improving metabolic health, and modulating the gut–brain axis and neurotransmitter regulation [43,44].”

**Modified text in manuscript (Implications and recommendations):**

“Our findings, together with the existing literature, suggest that diet quality may be a relevant factor to consider in the development of comprehensive student well-being strategies, including periodic mental health screening, nutritional counselling and education, and improved availability of healthy food in university cafeterias.”
