# Supplementary material for: Healthier dietary habits are associated with lower depression and anxiety among medical students at a private university in Lima, Peru: A cross-sectional study
Source: PLoS One. 2026 Jun 18;21(6):e0346062. doi: 10.1371/journal.pone.0346062 (PMC13278413; doi:10.1371/journal.pone.0346062)
Supplement: Peer review history

# Peer review: Decision Letter 0

**DOI:** 10.1371/journal.pone.0346062.r001

**Authors:** Yordanis Enríquez Canto

13 Apr 2026

PONE-D-26-09038

Healthier dietary habits are associated with lower depression and anxiety among medical students at a private university in Lima, Peru: A cross-sectional study

PLOS One

Dear Dr. Soriano-Moreno,

Thank you for submitting your manuscript to PLOS ONE. After careful consideration, we feel that it has merit but does not fully meet PLOS ONE’s publication criteria as it currently stands. Therefore, we invite you to submit a revised version of the manuscript that addresses the points raised during the review process.

Before addressing the specific revisions listed below, the authors should carefully review and respond to all comments provided by the reviewers. In summary, the main issues identified during peer review include:

- Insufficient methodological transparency, particularly regarding the selection of variables for bivariate and multivariable analyses, and the procedures used to assess and manage multicollinearity.
- Concerns about the dietary assessment instrument, as only 1.1% of participants were classified as having “healthy” dietary habits. This raises the possibility of misclassification and the need for stronger justification of the Spanish‑adapted Healthy Eating Index in this population.
- Overinterpretation of findings, including language suggesting a dose–response relationship or causal mechanisms that cannot be inferred from a cross-sectional design.
- Potential residual confounding, due to the absence of key variables such as sleep, physical activity, academic stress, socioeconomic status, and BMI in the adjusted models.
- Overreliance on p‑values, with a need to emphasize effect sizes and confidence intervals as the primary indicators of association.
- Limitations in generalizability, given the single‑center, convenience sample and the specific characteristics of the institutional context.
- The revised manuscript should address these issues comprehensively and ensure that all reviewer comments are incorporated into the response letter and the revised text.

Please submit your revised manuscript by May 28 2026 11:59PM. If you will need more time than this to complete your revisions, please reply to this message or contact the journal office at plosone@plos.org. When you're ready to submit your revision, log on to https://www.editorialmanager.com/pone/ and select the 'Submissions Needing Revision' folder to locate your manuscript file.

Please include the following items when submitting your revised manuscript:

- A letter that responds to each point raised by the academic editor and reviewer(s). You should upload this letter as a separate file labeled 'Response to Reviewers'.
- A marked-up copy of your manuscript that highlights changes made to the original version. You should upload this as a separate file labeled 'Revised Manuscript with Track Changes'.
- An unmarked version of your revised paper without tracked changes. You should upload this as a separate file labeled 'Manuscript'.

If you would like to make changes to your financial disclosure, please include your updated statement in your cover letter. Guidelines for resubmitting your figure files are available below the reviewer comments at the end of this letter.

If applicable, we recommend that you deposit your laboratory protocols in protocols.io to enhance the reproducibility of your results. Protocols.io assigns your protocol its own identifier (DOI) so that it can be cited independently in the future. For instructions see: https://journals.plos.org/plosone/s/submission-guidelines#loc-laboratory-protocols. Additionally, PLOS ONE offers an option for publishing peer-reviewed Lab Protocol articles, which describe protocols hosted on protocols.io. Read more information on sharing protocols at https://plos.org/protocols?utm_medium=editorial-email&utm_source=authorletters&utm_campaign=protocols.

As the corresponding author, your ORCID iD is verified in the submission system and will appear in the published article. PLOS supports the use of ORCID, and we encourage all coauthors to register for an ORCID iD and use it as well. Please encourage your coauthors to verify their ORCID iD within the submission system before final acceptance, as unverified ORCID iDs will not appear in the published article. Only the individual author can complete the verification step; PLOS staff cannot verify ORCID iDs on behalf of authors.

We look forward to receiving your revised manuscript.

Kind regards,

Yordanis Enríquez Canto, Ph.D.

Academic Editor

PLOS One

Journal Requirements:

When submitting your revision, we need you to address these additional requirements.

1. Please ensure that your manuscript meets PLOS ONE's style requirements, including those for file naming. The PLOS ONE style templates can be found at

https://journals.plos.org/plosone/s/file?id=wjVg/PLOSOne_formatting_sample_main_body.pdf and

https://journals.plos.org/plosone/s/file?id=ba62/PLOSOne_formatting_sample_title_authors_affiliations.pdf

2. Please include captions for your Supporting Information files at the end of your manuscript, and update any in-text citations to match accordingly. Please see our Supporting Information guidelines for more information: http://journals.plos.org/plosone/s/supporting-information.

3. Thank you for providing your underlying data as Supporting Information.

We note that the data set contains text or data that is not in English. Please note that PLOS is an English-language publisher, so we require data sets to be provided in English as well. Please upload an English-language version of your data set.

This will also allow us to determine if your data follows PLOS standards per our Data Availability policy here: https://journals.plos.org/plosone/s/data-availability

4. We note that there is identifying data in the Supporting Information file <Data.xlsx>. Due to the inclusion of these potentially identifying data, we have removed this file from your file inventory. Prior to sharing human research participant data, authors should consult with an ethics committee to ensure data are shared in accordance with participant consent and all applicable local laws.

Data sharing should never compromise participant privacy. It is therefore not appropriate to publicly share personally identifiable data on human research participants. The following are examples of data that should not be shared:

-Name, initials, physical address

-Ages more specific than whole numbers

-Internet protocol (IP) address

-Specific dates (birth dates, death dates, examination dates, etc.)

-Contact information such as phone number or email address

-Location data

-ID numbers that seem specific (long numbers, include initials, titled “Hospital ID”) rather than random (small numbers in numerical order)

Data that are not directly identifying may also be inappropriate to share, as in combination they can become identifying. For example, data collected from a small group of participants, vulnerable populations, or private groups should not be shared if they involve indirect identifiers (such as sex, ethnicity, location, etc.) that may risk the identification of study participants.

Additional guidance on preparing raw data for publication can be found in our Data Policy (https://journals.plos.org/plosone/s/data-availability#loc-human-research-participant-data-and-other-sensitive-data) and in the following article: http://www.bmj.com/content/340/bmj.c181.long.

Please remove or anonymize all personal information, ensure that the data shared are in accordance with participant consent, and re-upload a fully anonymized data set. Please note that spreadsheet columns with personal information must be removed and not hidden as all hidden columns will appear in the published file.

5. If the reviewer comments include a recommendation to cite specific previously published works, please review and evaluate these publications to determine whether they are relevant and should be cited. There is no requirement to cite these works unless the editor has indicated otherwise.

**Additional Editor Comments:**

After careful evaluation of the manuscript and consideration of the reviewers’ comments, I believe your study addresses an important topic and provides potentially valuable evidence regarding dietary habits and mental health among medical students in Peru. However, both reviewers identified methodological and interpretive issues that must be addressed before the manuscript can be considered for publication.

On this basis, I invite you to submit a revised version of your manuscript. The revision should address all required points listed below. I distinguish clearly between required and recommended changes, and I provide editorial guidance where reviewer comments diverge.

1. Required Revisions

1.1. Clarify and justify the analytical approach

Both reviewers noted insufficient transparency regarding the selection of variables for bivariate and multivariable analyses. The manuscript should:

Provide a clear description of the variable selection strategy for regression models (e.g., theoretical criteria, epidemiological relevance, or statistical thresholds).

Explain how multicollinearity was assessed and handled. The manuscript mentions categorizing age into tertiles due to multicollinearity, but the diagnostics (e.g., VIF values or correlation matrices) are not described.

Report goodness‑of‑fit measures appropriate for Poisson regression with robust variance.

These additions are essential for evaluating the robustness of your findings.

1.2. Address concerns regarding the dietary assessment instrument

Reviewer 2 raised a substantive concern: only 1.1% of participants were classified as having “healthy” dietary habits. This extremely low proportion suggests potential misclassification or lack of calibration of the Spanish‑adapted Healthy Eating Index (HEI) for this population.

Please:

Provide a stronger justification for using the Spanish-adapted HEI in Peruvian medical students.

Discuss explicitly the possibility of exposure misclassification, including how cultural and dietary differences may affect scoring.

Consider conducting a sensitivity analysis (e.g., tertiles or quartiles of HEI score) to evaluate whether the association remains consistent under alternative categorizations.

1.3. Revise interpretation to avoid causal implications

The manuscript currently suggests a “dose–response effect” and discusses biological mechanisms in a way that implies causality. Given the cross-sectional design:

Remove or rephrase any language implying temporal ordering or causal inference.

Expand the discussion of reverse causation, acknowledging that mental health symptoms may influence dietary behavior.

Ensure that biological mechanisms are presented as hypothetical and not as explanations supported by the present study.

1.4. Discuss unmeasured confounding

The adjusted models do not include several important confounders (e.g., sleep quality, physical activity, academic stress, socioeconomic status, BMI). While these variables may not have been collected:

Provide a clear justification for their absence.

Expand the discussion of residual confounding and how it may influence the observed associations.

1.5. Emphasize effect sizes and confidence intervals

Reviewer 1 recommends reducing reliance on p‑values. Please:

Highlight prevalence ratios and confidence intervals as the primary indicators of association.

Avoid interpreting results solely based on statistical significance.

2. Recommended Revisions (Not Required but Strongly Encouraged)

Improve the clarity and flow of the writing, particularly in the Introduction and Discussion, to enhance readability.

Expand the discussion of generalizability, considering the single‑center, convenience sample and the unique characteristics of a Seventh‑day Adventist institution.

Provide additional descriptive insights into dietary patterns if the dataset allows (e.g., distribution of HEI components).

Consider adding a conceptual diagram illustrating potential pathways and confounders.

3. Editorial Notes

After reviewing the manuscript in detail, I also recommend:

Clarifying the data collection period in relation to the academic calendar, as mental health symptoms may vary across semesters.

Ensuring consistency in reporting (e.g., whether HEI is treated as continuous, categorical, or both).

[Note: HTML markup is below. Please do not edit.]

Reviewers' comments:

Reviewer's Responses to Questions

**Comments to the Author**

1. Is the manuscript technically sound, and do the data support the conclusions?

The manuscript must describe a technically sound piece of scientific research with data that supports the conclusions. Experiments must have been conducted rigorously, with appropriate controls, replication, and sample sizes. The conclusions must be drawn appropriately based on the data presented.

Reviewer #1: Yes

Reviewer #2: Partly

**********

2. Has the statistical analysis been performed appropriately and rigorously?

Reviewer #1: Yes

Reviewer #2: No

**********

3. Have the authors made all data underlying the findings in their manuscript fully available?

The PLOS Data policy requires authors to make all data underlying the findings described in their manuscript fully available without restriction, with rare exception (please refer to the Data Availability Statement in the manuscript PDF file). The data should be provided as part of the manuscript or its supporting information, or deposited to a public repository. For example, in addition to summary statistics, the data points behind means, medians and variance measures should be available. If there are restrictions on publicly sharing data—e.g. participant privacy or use of data from a third party—those must be specified.

Reviewer #1: Yes

Reviewer #2: Yes

**********

4. Is the manuscript presented in an intelligible fashion and written in standard English?

PLOS ONE does not copyedit accepted manuscripts, so the language in submitted articles must be clear, correct, and unambiguous. Any typographical or grammatical errors should be corrected at revision, so please note any specific errors here.

Reviewer #1: Yes

Reviewer #2: Yes

**********

5. Review Comments to the Author

Please use the space provided to explain your answers to the questions above. You may also include additional comments for the author, including concerns about dual publication, research ethics, or publication ethics. (Please upload your review as an attachment if it exceeds 20,000 characters)

Reviewer #1: The paper and its findings are interesting, and it is commendable that all data have been made available so that interested readers can verify the results and even conduct additional analyses if desired.

The analytical methods are primarily exploratory in nature, which is appropriate given that a convenience sample was used. In addition to simple bivariate associations, Poisson regression and adjusted regression models have been applied.

However, there are several areas that would benefit from further clarification and improvement. The original dataset contains more than 30 variables, yet it is unclear how variables were selected for inclusion in the bivariate analyses and subsequent regression models. A systematic description of this variable selection process is needed.

Furthermore, the manuscript does not address how correlations among independent variables were handled in the regression analyses. If multicollinearity is present, the authors should describe the steps taken to assess and mitigate its impact (e.g., variable selection procedures, variance inflation factors, or other diagnostics).

For all regression models, appropriate goodness-of-fit measures should be reported to help readers evaluate model adequacy.

Additionally, reliance on p-values is not ideal in the context of exploratory analysis. Instead, the authors are encouraged to emphasize effect sizes along with 95% or 99% confidence intervals, which provide more informative measures of uncertainty.

Overall, this is an interesting and potentially valuable paper, but greater methodological transparency would strengthen its rigor and reproducibility.

Reviewer #2: This manuscript examines the association between dietary habits and symptoms of anxiety and depression in medical students; however, in its current form, it presents substantial methodological and interpretive limitations that weaken the validity of its conclusions. While the use of validated screening tools (PHQ-9, GAD-7) and prevalence ratios is appropriate, the reliance on a single-centre, convenience sample from a private Adventist university severely limits external validity and introduces potential selection bias. More importantly, the measurement of the exposure is problematic. Only 1.1% of participants were classified as having “healthy” dietary habits, which suggests a lack of calibration of the instrument for this population. The use of a Spanish-adapted Healthy Eating Index, based on dietary recommendations from the Spanish context, may not be adequately justified for Peruvian medical students and may not reflect local dietary patterns. This raises concern that the instrument is misclassifying participants, thereby undermining the validity and interpretability of the exposure itself.

The manuscript also overreaches in its interpretation. The authors invoke a “dose-response effect” under the Limitations and Strengths section and suggest that the observed gradient supports a potential causal relationship, which is not justified given the cross-sectional design. Temporal ambiguity and reverse causation remain unresolved, particularly because mental health status may influence dietary behaviour. In addition, the adjusted models omit several key confounders, including sleep, physical activity, academic stress, socioeconomic status, and BMI, increasing the likelihood of residual confounding. The discussion further extends beyond the evidence by invoking biological mechanisms and policy implications that are not supported by the study design. Taken together, these issues indicate that the manuscript, in its current form, does not meet the standard of analytical rigour expected for publication. I recommend major revision, with substantial revision of the interpretation, explicit justification or reconsideration of the dietary measurement approach, and a more rigorous treatment of the study’s limitations.

**********

6. PLOS authors have the option to publish the peer review history of their article (what does this mean?). If published, this will include your full peer review and any attached files.

If you choose “no”, your identity will remain anonymous but your review may still be made public.

**Do you want your identity to be public for this peer review?**   For information about this choice, including consent withdrawal, please see our Privacy Policy.

Reviewer #1: No

Reviewer #2: No

**********

[NOTE: If reviewer comments were submitted as an attachment file, they will be attached to this email and accessible via the submission site. Please log into your account, locate the manuscript record, and check for the action link "View Attachments". If this link does not appear, there are no attachment files.]

To ensure your figures meet our technical requirements, please review our figure guidelines: https://journals.plos.org/plosone/s/figures

You may also use PLOS’s free figure tool, NAAS, to help you prepare publication quality figures: https://journals.plos.org/plosone/s/figures#loc-tools-for-figure-preparation.

NAAS will assess whether your figures meet our technical requirements by comparing each figure against our figure specifications.

# Author comment: Author response to Decision Letter 1

**DOI:** 10.1371/journal.pone.0346062.r002

20 Apr 2026

RESPONSE TO REVIEWERS

Manuscript title: Healthier dietary habits are associated with lower depression and anxiety among medical students at a private university in Lima, Peru: A cross-sectional study

Manuscript ID: PONE-D-26-09038

We sincerely thank the Academic Editor, and both reviewers for their thorough, rigorous, and constructive evaluation of our manuscript. Their comments identified areas requiring greater methodological transparency, more cautious interpretation of findings, and more explicit discussion of the study’s limitations. We have addressed each concern systematically in the response below, and we believe the revised manuscript is substantially stronger because of this process.

ACADEMIC EDITOR COMMENTS

Comment AE 1.1. “Clarify and justify the analytical approach. Provide a clear description of the variable selection strategy for regression models (e.g., theoretical criteria, epidemiological relevance, or statistical thresholds). Explain how multicollinearity was assessed and handled—the manuscript mentions categorizing age into tertiles due to multicollinearity, but the diagnostics are not described. Report goodness-of-fit measures appropriate for Poisson regression with robust variance.”

Response: We appreciate this important observation. Covariates for the adjusted regression models were selected a priori on the basis of theoretical association with the outcomes and epidemiological relevance, rather than on statistical cut-offs such as p-values in the bivariate analysis. We recognize that this strategy was not sufficiently described in the original manuscript and have expanded the Statistical analysis section accordingly, adding references supporting the selection of each covariate. Multicollinearity among the independent variables included in each adjusted model was formally evaluated using the variance inflation factor (VIF). When age was initially entered as a continuous variable, a VIF of 25.7 was observed, indicating severe multicollinearity. To address this, age was recategorized into tertiles and included in this categorical form in the adjusted models, after which all VIF values were below the conventional threshold of 10. Regarding goodness-of-fit, we respectfully note that the aim of our analysis was etiologic/associational rather than predictive. Consequently, the regression models were not intended to maximize predictive performance but to estimate the association between dietary habits and the prevalence of anxiety and depression while adjusting for theoretically relevant confounders. For this reason, goodness-of-fit indices were not a primary focus of the analysis. We have clarified this analytical purpose in the revised Methods.

Modified text in manuscript (Methods – Statistical analysis):

“The association between the Healthy Eating Index score and the prevalence of depression or anxiety was evaluated using Poisson regression with robust variance, estimating crude (cPR) and adjusted prevalence ratios (aPR) with 95% confidence intervals (95% CI). Variables considered potential confounders based on theoretical and epidemiological criteria were included in the adjusted model [31–33]. Marital status was not included in the adjusted model because it had very few observations in some categories, and most participants were single. Multicollinearity among the independent variables was formally assessed using the variance inflation factor (VIF). When age was initially modelled as a continuous variable, a VIF of 25.7 was observed, indicating severe collinearity; therefore, age was recategorized into tertiles for inclusion in the adjusted analyses, after which all VIF values were below 10.”

Comment AE 1.2. “Address concerns regarding the dietary assessment instrument. Only 1.1% of participants were classified as having ‘healthy’ dietary habits. This extremely low proportion suggests potential misclassification or lack of calibration of the Spanish-adapted HEI for this population. Provide a stronger justification for using the Spanish-adapted HEI; discuss exposure misclassification including how cultural and dietary differences may affect scoring; consider a sensitivity analysis using tertiles or quartiles of HEI score.”

Response: We fully agree with the Academic Editor that the Spanish-adapted HEI provides categorical cut-offs that have not been standardized or formally calibrated for the Peruvian population, and this represents an important limitation of the instrument. For this reason, the three categories (unhealthy, needs improvement, healthy) were reported descriptively only, to characterize the sample and to enable comparison with previous literature. Crucially, the regression models estimating the association between dietary habits and anxiety/depression were not based on these categories but on the HEI total score modelled as a continuous variable. This analytical choice avoids reliance on cut-offs that have not been validated in Peruvian youth and preserves the full informational content of the scale. For the same reason, we respectfully consider that a complementary analysis using tertiles or quartiles of the HEI score would not add substantive information beyond what the linear model and its post-estimation plots already convey and could in fact reintroduce arbitrary categorizations of the exposure. We selected the Spanish-adapted HEI because it is the best-documented instrument available in Spanish for quantitative assessment of overall diet quality, and because the dietary recommendations underlying its scoring are closer to the Peruvian dietary context than those of purely Anglo-American instruments. We have added a clear acknowledgement of exposure misclassification and of the lack of Peruvian calibration of the categorical cut-offs to the Methods and the Limitations section.

Modified text in manuscript (Methods – Variables):

“The total score ranged from 0 to 100. Final scores were categorized as: >80 (“healthy”), 50–80 (“needs improvement”), and <50 (“unhealthy”). Nevertheless, these cut-off points should be interpreted cautiously, as they have not been specifically validated in Peruvian populations or among university students.”

Modified text in manuscript (Limitations and strengths):

“Also, the categorical cut-offs proposed for the Spanish-adapted HEI have not been standardized for the Peruvian population. This may help explain the very low proportion of students classified as having ‘healthy’ dietary habits (1.1%).”

Comment AE 1.3. “Revise interpretation to avoid causal implications. The manuscript suggests a ‘dose–response effect’ and discusses biological mechanisms in a way that implies causality. Remove or rephrase language implying temporal ordering or causal inference; expand the discussion of reverse causation; ensure biological mechanisms are presented as hypothetical.”

Response: We agree and have carefully revised the language throughout the manuscript. The term “dose–response effect” has been removed and replaced, where appropriate, with “inverse linear relationship” or “inversely proportional relationship” between the healthy eating score and the prevalence of anxiety and depression, which better reflects the statistical behaviour of the association. Statements that could imply temporal ordering or causal inference have been softened or removed. Biological mechanisms are now explicitly framed as hypothetical explanations supported by previous literature rather than as findings of the present study. Furthermore, we have expanded the discussion of reverse causation, acknowledging that symptoms of anxiety and depression may themselves modify dietary behaviour.

Modified text in manuscript (Limitations and strengths):

“Second, the cross-sectional design precludes establishing temporality and directionality between dietary habits and symptoms of anxiety and depression, hence reverse causality is plausible, as symptoms of depression and anxiety are known to alter appetite, food preferences, and eating behavior, which may result in poorer diet quality.”

“In addition, the strength of the association and the inversely proportional relationship supports a potential association between the variables.”

Modified text in manuscript (Discussion – second paragraph on mechanisms):

“In this context, plausible biological pathways proposed in the literature suggest that healthy dietary patterns may protect against depression and anxiety by reducing inflammation and oxidative stress, improving metabolic health, and modulating the gut–brain axis and neurotransmitter regulation [43,44]. On the other hand, high consumption of ultra-processed foods may contribute to worsening mental health by promoting inflammation, dysregulation of the hypothalamic–pituitary–adrenal axis, and alterations in the synthesis of serotonin, dopamine, and norepinephrine [14].”

Comment AE 1.4. “Discuss unmeasured confounding. The adjusted models do not include several important confounders (sleep quality, physical activity, academic stress, socioeconomic status, BMI). Provide a clear justification for their absence and expand the discussion of residual confounding.”

Response: We thank the Academic Editor for this well-taken observation. We acknowledge that sleep quality, physical activity, academic stress, and socioeconomic status were not collected in this study and therefore could not be included in the adjusted models, which is a genuine limitation that we have added explicitly to the Limitations section. We also note that residual confounding by the variables mentioned by the Academic Editor may have led to either underestimation or overestimation of the observed association. As is often the case, adjustment for additional correlated lifestyle confounders (e.g., physical activity, sleep) might move the effect estimate somewhat toward the null, though the direction and magnitude cannot be anticipated with certainty. An important distinction that we would like to raise is that body mass index (BMI) is most plausibly a mediator rather than a confounder of the relationship between dietary habits and mental health, since diet quality is an established determinant of BMI, and BMI itself has been linked to depressive and anxiety symptoms. Adjusting for BMI could therefore produce overadjustment bias and partially block the total effect of diet quality on mental health. For these reasons, BMI would not have been included in the adjusted model even if it had been available. These considerations have been added to the revised Discussion.

Modified text in manuscript (Limitations and strengths):

“In addition, residual confounding is an important limitation. Variables associated with both dietary habits and mental health in university students, such as sleep quality, physical activity, academic stress, and socioeconomic status, were not measured and therefore could not be included in the adjusted models. This may have led to underestimation or overestimation of the observed associations.”

Comment AE 1.5. “Emphasize effect sizes and confidence intervals. Highlight prevalence ratios and confidence intervals as the primary indicators of association; avoid interpreting results solely based on statistical significance.”

Response: We fully agree. The Results and Discussion have been revised to place the adjusted prevalence ratios and their 95% confidence intervals at the center of interpretation, rather than focusing on p-values alone. To further reinforce the robustness and precision of the estimates, we added a sensitivity analysis using 99% confidence intervals. The results remained consistent: anxiety aPR = 0.97 (99% CI: 0.95 to 0.98; p<0.001) and depression aPR = 0.98 (99% CI: 0.96 to 0.99; p<0.001). We have added this sensitivity analysis to the Methods, Results, and Discussion sections, and we have reframed the interpretation of the findings around effect size and precision. We wish to note, however, that we have been careful not to overclaim certainty or causality on the basis of these findings.

Modified text in manuscript (Methods – Statistical analysis):

“As a sensitivity analysis, the same adjusted Poisson regression models were refitted using 99% confidence intervals (99% CI) in order to evaluate the robustness of the estimated associations under a more conservative precision threshold.”

Modified text in manuscript (Results):

“In the sensitivity analysis using 99% CIs, the estimates remained highly statistically significant (anxiety: aPR: 0.97; 99% CI: 0.95 to 0.98; p<0.001; depression: aPR: 0.98; 99% CI: 0.96 to 0.99; p<0.001).”

Modified text in manuscript (Discussion – Main findings):

“In our study, this association was also supported by the strength and consistency of the findings, as each additional point in the HEI was associated with a 3% lower prevalence of anxiety, and this association remained highly statistically significant in the sensitivity analysis using 99% CI.”

Comment AE 2.1. “[Recommended] Expand the discussion of generalizability, considering the single-center, convenience sample and the unique characteristics of a Seventh-day Adventist institution.”

Response: We agree and have expanded the discussion of external validity in the Limitations and in the Implications and recommendations sections. The study was conducted at a single institution, with non-probabilistic convenience sampling, in medical students from a university sponsored by the Seventh-day Adventist Church. The Adventist tradition explicitly promotes a predominantly plant-based dietary pattern, regular physical activity, and abstinence from alcohol and tobacco as part of its health message.

Modified text in manuscript (Implications and recommendations):

“Our findings may be generalizable to similar university settings where medical students facing high academic demands exhibit a high prevalence of depressive (45.1%) and anxiety (34.9%) symptoms, along with a high frequency of dietary patterns that “need improvement” (77.3%) or are considered “unhealthy” (21.6%). However, the study context should be considered when interpreting external validity. This university is sponsored by the Seventh-day Adventist Church, which promotes a predominantly plant-based diet, regular physical activity, and abstinence from alcohol and tobacco. As a result, students may be influenced by institutional, religious, and peer-related factors that shape dietary habits differently from those in secular universities.”

Comment AE 3.1. “[Editorial Note] Clarify the data collection period in relation to the academic calendar, as mental health symptoms may vary across semesters.”

Response: We agree and have clarified the data collection period and its position within the academic calendar in the Methods section. We have also noted that the window included the end-of-semester examination phase, which is typically associated with heightened academic stress and may influence both dietary behavior and mental health symptom burden.

Modified text in manuscript (Methods – Procedures):

“Data collection was conducted from 15/07/2025 to 15/12/2025, corresponding to the second academic semester at Universidad Peruana Unión. This period includes regular academic activities and the end-of-semester examination phase, during which students are often exposed to constant evaluations and sustained academic stress, both of which may influence dietary behaviors and mental health symptom burden.”

Comment AE 3.2. “[Editorial Note] Ensure consistency in reporting (e.g., whether HEI is treated as continuous, categorical, or both).”

Response: We thank the Academic Editor for this helpful observation. We have revised the manuscript to ensure consistency throughout. Specifically, we now state explicitly that: the HEI was used categorically for descriptive purposes only.

Modified text in manuscript (Methods – Statistical analysis):

“The categorization of the HEI score as unhealthy, needs improvement, and healthy was applied only for descriptive analyses.”

REVIEWER #1 COMMENTS

Comment R1.1. “The original dataset contains more than 30 variables, yet it is unclear how variables were selected for inclusion in the bivariate analyses and subsequent regression models. A systematic description of this variable selection process is needed.”

Response: We thank the Reviewer for highlighting this point. Most of t

Attachment Submitted filename: Response to reviewers.docx

# Peer review: Decision Letter 1

**DOI:** 10.1371/journal.pone.0346062.r003

**Authors:** Yordanis Enríquez Canto

13 May 2026

PONE-D-26-09038R1

Healthier dietary habits are associated with lower depression and anxiety among medical students at a private university in Lima, Peru: A cross-sectional study

PLOS One

Dear Dr. Soriano-Moreno,

Thank you for submitting your manuscript to PLOS ONE. After careful consideration, we feel that it has merit but does not fully meet PLOS ONE’s publication criteria as it currently stands. Therefore, we invite you to submit a revised version of the manuscript that addresses the points raised during the review process.

While your revised manuscript addresses most of the concerns raised previously, two key issues remain. First, you are asked to provide a stronger justification for the choice of dietary assessment tool and to perform a sensitivity analysis with an alternative exposure specification. Second, the limitations section should more explicitly address potential residual confounding related to academic stress and socioeconomic status, especially during the examination period. Please refer to the reviewer’s detailed comments below.

Please submit your revised manuscript by Jun 27 2026 11:59PM. If you will need more time than this to complete your revisions, please reply to this message or contact the journal office at plosone@plos.org. When you're ready to submit your revision, log on to https://www.editorialmanager.com/pone/ and select the 'Submissions Needing Revision' folder to locate your manuscript file.

Please include the following items when submitting your revised manuscript:

- A letter that responds to each point raised by the academic editor and reviewer(s). You should upload this letter as a separate file labeled 'Response to Reviewers'.
- A marked-up copy of your manuscript that highlights changes made to the original version. You should upload this as a separate file labeled 'Revised Manuscript with Track Changes'.
- An unmarked version of your revised paper without tracked changes. You should upload this as a separate file labeled 'Manuscript'.

If you would like to make changes to your financial disclosure, please include your updated statement in your cover letter. Guidelines for resubmitting your figure files are available below the reviewer comments at the end of this letter.

If applicable, we recommend that you deposit your laboratory protocols in protocols.io to enhance the reproducibility of your results. Protocols.io assigns your protocol its own identifier (DOI) so that it can be cited independently in the future. For instructions see: https://journals.plos.org/plosone/s/submission-guidelines#loc-laboratory-protocols. Additionally, PLOS ONE offers an option for publishing peer-reviewed Lab Protocol articles, which describe protocols hosted on protocols.io. Read more information on sharing protocols at https://plos.org/protocols?utm_medium=editorial-email&utm_source=authorletters&utm_campaign=protocols.

As the corresponding author, your ORCID iD is verified in the submission system and will appear in the published article. PLOS supports the use of ORCID, and we encourage all coauthors to register for an ORCID iD and use it as well. Please encourage your coauthors to verify their ORCID iD within the submission system before final acceptance, as unverified ORCID iDs will not appear in the published article. *Only*   the individual author can complete the verification step; PLOS staff *cannot*   verify ORCID iDs on behalf of authors.

We look forward to receiving your revised manuscript.

Kind regards,

Yordanis Enríquez Canto, Ph.D.

Academic Editor

PLOS One

Journal Requirements:

If the reviewer comments include a recommendation to cite specific previously published works, please review and evaluate these publications to determine whether they are relevant and should be cited. There is no requirement to cite these works unless the editor has indicated otherwise.

Please review your reference list to ensure that it is complete and correct. If you have cited papers that have been retracted, please include the rationale for doing so in the manuscript text, or remove these references and replace them with relevant current references. Any changes to the reference list should be mentioned in the rebuttal letter that accompanies your revised manuscript. If you need to cite a retracted article, indicate the article’s retracted status in the References list and also include a citation and full reference for the retraction notice.

Additional Editor Comments:

Thank you for your careful and thorough response to the issues raised in the first round of review. Reviewer 2 acknowledges significant improvements to the manuscript, particularly regarding external validity, causal interpretation, reverse causation, and the discussion of biological mechanisms and practical implications. The clarification of the institutional context and the use of the HEI score as a continuous variable are also noted as strengths.

However, two main points require further attention:

Justification and Sensitivity of the Dietary Instrument:

While the usage of the Spanish-adapted HEI is now more clearly explained, Reviewer 2 notes that linguistic adaptation does not necessarily equate to cultural or dietary suitability for the Peruvian context. Please clarify whether other Latin American adaptations of the HEI (such as the Brazilian version) were considered, and provide a rationale for selecting the Spanish-adapted version. In addition, please conduct a sensitivity analysis with an alternative specification of the dietary exposure variable—such as a flexible approach (e.g., restricted cubic splines) or a categorization based on the sample distribution—to examine whether the association is robust beyond the assumed linear relationship.

Residual Confounding:

While the revised manuscript acknowledges residual confounding and reverse causality, we recommend making the limitation more explicit, particularly with respect to academic stress and socioeconomic status during the end-of-semester examination period. These factors could plausibly influence both diet quality and anxiety/depressive symptoms and were not measured. Please add a statement clarifying that the observed association may in part reflect broader academic and socioeconomic conditions.

[Note: HTML markup is below. Please do not edit.]

Reviewers' comments:

Reviewer's Responses to Questions

**Comments to the Author**

1. If the authors have adequately addressed your comments raised in a previous round of review and you feel that this manuscript is now acceptable for publication, you may indicate that here to bypass the “Comments to the Author” section, enter your conflict of interest statement in the “Confidential to Editor” section, and submit your "Accept" recommendation.

Reviewer #2: All comments have been addressed

**********

2. Is the manuscript technically sound, and do the data support the conclusions?

The manuscript must describe a technically sound piece of scientific research with data that supports the conclusions. Experiments must have been conducted rigorously, with appropriate controls, replication, and sample sizes. The conclusions must be drawn appropriately based on the data presented.

Reviewer #2: Yes

**********

3. Has the statistical analysis been performed appropriately and rigorously?

Reviewer #2: Yes

**********

4. Have the authors made all data underlying the findings in their manuscript fully available?

The PLOS Data policy requires authors to make all data underlying the findings described in their manuscript fully available without restriction, with rare exception (please refer to the Data Availability Statement in the manuscript PDF file). The data should be provided as part of the manuscript or its supporting information, or deposited to a public repository. For example, in addition to summary statistics, the data points behind means, medians and variance measures should be available. If there are restrictions on publicly sharing data—e.g. participant privacy or use of data from a third party—those must be specified.

Reviewer #2: Yes

**********

5. Is the manuscript presented in an intelligible fashion and written in standard English?

PLOS ONE does not copyedit accepted manuscripts, so the language in submitted articles must be clear, correct, and unambiguous. Any typographical or grammatical errors should be corrected at revision, so please note any specific errors here.

Reviewer #2: Yes

**********

6. Review Comments to the Author

Please use the space provided to explain your answers to the questions above. You may also include additional comments for the author, including concerns about dual publication, research ethics, or publication ethics. (Please upload your review as an attachment if it exceeds 20,000 characters)

Reviewer #2: The authors have responded seriously to the concerns raised in the first round, and several of the revisions improve the manuscript. I consider the concerns regarding external validity, causal overinterpretation, reverse causation, and the discussion of biological mechanisms and practical implications to be largely addressed. In particular, the manuscript now better acknowledges the single-centre Adventist institutional context, removes the previous "dose-response" wording, explicitly discusses reverse causation, and frames biological mechanisms and practical implications more cautiously.

The authors have adequately addressed my concern regarding external validity. The revised text appropriately frames the Adventist affiliation as part of the institutional context, without assuming students’ individual religious affiliation or adherence to Adventist practices. I do not request further revisions on this point.

My main remaining concern relates to the dietary exposure measure. The authors have improved the manuscript by clarifying that the HEI categories were used only descriptively and that the regression models used the total HEI score as a continuous variable. They also appropriately acknowledge that the categorical cut-offs have not been validated in Peruvian populations and that this may help explain the very low proportion of participants classified as having "healthy" dietary habits. However, the concern is not limited to the cut-offs, but also to the contextual validity of the dietary score itself. The justification for using the Spanish-adapted HEI remains somewhat limited, since linguistic availability does not necessarily imply dietary or cultural suitability for Peru. I recommend that the authors clarify whether other Latin American adaptations of the HEI, including Brazilian adaptations, were considered, and explain why the Spanish-adapted instrument was preferred.

In addition, although I understand the authors’ concern that tertiles or quartiles may introduce arbitrary categorisation, an additional sensitivity analysis using an alternative exposure specification would strengthen the manuscript. This need not be limited to tertiles or quartiles. A flexible modelling approach, such as restricted cubic splines, or a sample-distribution-based categorisation, could help assess whether the association depends on assuming a linear relationship across the HEI score range.

I appreciate that the authors now acknowledge residual confounding and reverse causality. However, given that the data collection period included the end-of-semester examination phase, a period in which academic stress, sleep disruption, reduced physical activity, reduced time for meal preparation, and poorer mental health symptoms may co-occur, I recommend making the residual confounding limitation slightly more explicit with regard to academic stress and socioeconomic status. These factors may plausibly affect both diet quality and anxiety/depressive symptoms. The manuscript should therefore make clear that the observed association may partly reflect broader academic and socioeconomic conditions that were not measured.

Overall, the manuscript has improved, and several of my initial concerns have been adequately addressed. I recommend further revision focusing on two remaining issues: first, a stronger justification and sensitivity assessment of the dietary exposure measure; second, a more explicit treatment of residual confounding, especially in relation to the examination period and socioeconomic status.

**********

7. PLOS authors have the option to publish the peer review history of their article (what does this mean?). If published, this will include your full peer review and any attached files.

If you choose “no”, your identity will remain anonymous but your review may still be made public.

**Do you want your identity to be public for this peer review?**   For information about this choice, including consent withdrawal, please see our Privacy Policy.

Reviewer #2: No

**********

[NOTE: If reviewer comments were submitted as an attachment file, they will be attached to this email and accessible via the submission site. Please log into your account, locate the manuscript record, and check for the action link "View Attachments". If this link does not appear, there are no attachment files.]

To ensure your figures meet our technical requirements, please review our figure guidelines: https://journals.plos.org/plosone/s/figures

You may also use PLOS’s free figure tool, NAAS, to help you prepare publication quality figures: https://journals.plos.org/plosone/s/figures#loc-tools-for-figure-preparation.

NAAS will assess whether your figures meet our technical requirements by comparing each figure against our figure specifications.

# Author comment: Author response to Decision Letter 2

**DOI:** 10.1371/journal.pone.0346062.r004

18 May 2026

RESPONSE TO REVIEWERS

Manuscript title: Healthier dietary habits are associated with lower depression and anxiety among medical students at a private university in Lima, Peru: A cross-sectional study

Manuscript ID: PONE-D-26-09038

We sincerely thank the Academic Editor and Reviewer 2 for their prompt response and for the thorough, rigorous, and constructive evaluation of our manuscript. Their comments identified areas requiring greater methodological transparency, more cautious interpretation of findings, and a more explicit discussion of the study’s limitations. We have addressed each concern systematically in the response below, and we believe the revised manuscript is substantially stronger because of this process.

REVIEWER #2 COMMENTS

Comment R2.1. “My main remaining concern relates to the dietary exposure measure. The authors have improved the manuscript by clarifying that the HEI categories were used only descriptively and that the regression models used the total HEI score as a continuous variable. They also appropriately acknowledge that the categorical cut-offs have not been validated in Peruvian populations and that this may help explain the very low proportion of participants classified as having "healthy" dietary habits. However, the concern is not limited to the cut-offs, but also to the contextual validity of the dietary score itself. The justification for using the Spanish-adapted HEI remains somewhat limited, since linguistic availability does not necessarily imply dietary or cultural suitability for Peru. I recommend that the authors clarify whether other Latin American adaptations of the HEI, including Brazilian adaptations, were considered, and explain why the Spanish-adapted instrument was preferred.”

Response: We thank the reviewer for this important observation. We agree that the concern is not limited to the categorical cut-offs, but also relates to the contextual validity of the dietary score itself. Therefore, we have revised the Methods, Discussion, and Limitations sections to clarify this issue.

In the Methods section, we now explain that the Spanish-adapted HEI was selected because, to our knowledge, no HEI adaptation has been validated specifically for the Peruvian population or for Peruvian university students. Although originally developed in Spain, this instrument was considered suitable for our questionnaire-based study because it is available in Spanish, uses broad and easily understandable food groups, and includes dietary domains that are broadly consistent with the Peruvian Dietary Guidelines, including fruits, vegetables, legumes, dairy products, animal-source foods, sweets, sugar-sweetened beverages, and processed meats. We also expanded the Discussion to contextualize the low proportion of students classified as having healthy dietary habits. We now mention that similarly low frequencies have been reported in previous Latin American studies, including 0.8% in an adult population from urban Huancavelica, Peru, 9.3% among Chilean university students, and 1% among Mexican adolescent women. Therefore, the low frequency observed in our study (1.1%) does not appear to be an isolated finding, but rather is consistent with previous regional evidence. We also discuss that this may reflect the difficulty of meeting strict healthy eating thresholds in real-world student populations, irregular eating schedules, academic stress, limited time for meal planning, and high availability of ultra-processed foods and sugar-sweetened beverages. Finally, we strengthened the Limitations.

Modified text in Methods: “Healthy dietary habits were assessed using the Spanish-adapted Healthy Eating Index (HEI) [28]. This instrument was selected because no HEI adaptation has been validated specifically for the Peruvian population. The Spanish-adapted HEI was considered suitable for this study because it is available in Spanish, uses broad food groups that are understandable for Peruvian students, and includes dietary domains that are broadly consistent with the Peruvian Dietary Guidelines, such as fruits, vegetables, legumes, dairy products, animal-source foods, sweets, sugar-sweetened beverages, and processed meats [29].”

Added text in Discussion: “The low proportion of students classified as having healthy dietary habits is consistent with previous Latin American evidence. In Peru, a study from Huancavelica using the HEI found that only 0.8% of adults had a healthy diet [41]. Similarly, among Chilean university students, only 9.3% had a healthy diet, while 55.3% needed changes and 35.4% had an unhealthy diet [42]. In Mexico, only 1% of adolescent women were classified as having good diet quality using an adapted HEI [43]. This may reflect the difficulty of meeting strict healthy eating thresholds in real-world student populations, as well as irregular eating schedules, academic stress, limited time for meal planning, and high availability of ultra-processed foods and sugar-sweetened beverages.”

Added text in Limitations: “Although validated instruments were used, the PHQ-9 and GAD-7 do not replace a clinical reference diagnosis. Regarding dietary exposure, the Spanish-adapted HEI has not been culturally validated in Peru or among Peruvian university students, which may have introduced non-differential exposure misclassification because it may not fully capture local dietary patterns, portion sizes, cooking methods, sodium intake, or degree of food processing. Also, its categorical cut-offs have not been standardized for the Peruvian population, which may explain the very low proportion of students classified as having ‘healthy’ dietary habits (1.1%), although this finding is consistent with previous Latin American evidence.”

Comment R2.2. “In addition, although I understand the authors’ concern that tertiles or quartiles may introduce arbitrary categorisation, an additional sensitivity analysis using an alternative exposure specification would strengthen the manuscript. This need not be limited to tertiles or quartiles. A flexible modelling approach, such as restricted cubic splines, or a sample-distribution-based categorisation, could help assess whether the association depends on assuming a linear relationship across the HEI score range.”

Response: We thank the reviewer for this helpful suggestion. We agree that alternative exposure specifications may be useful when the association between a continuous exposure and the outcome is non-linear. However, in our study, the linearity assumption between the HEI score and each outcome was assessed before fitting the regression models using the “lincheck” command, showing an approximately linear inverse relationship with both anxiety and depression. Therefore, we considered the continuous linear specification appropriate. Using restricted cubic splines would have added unnecessary complexity without improving interpretability.

Added text in Methods, Statistical analysis: “Before fitting the regression models, the linearity assumption between the HEI score and each outcome was assessed using the “lincheck” command.”

Comment R2.3. “I appreciate that the authors now acknowledge residual confounding and reverse causality. However, given that the data collection period included the end-of-semester examination phase, a period in which academic stress, sleep disruption, reduced physical activity, reduced time for meal preparation, and poorer mental health symptoms may co-occur, I recommend making the residual confounding limitation slightly more explicit with regard to academic stress and socioeconomic status. These factors may plausibly affect both diet quality and anxiety/depressive symptoms. The manuscript should therefore make clear that the observed association may partly reflect broader academic and socioeconomic conditions that were not measured.”

Response: We thank the reviewer for this helpful comment. We agree that residual confounding should be described more explicitly, particularly because data collection included the end-of-semester examination phase. We have revised the Limitations section to clarify that unmeasured factors such as academic stress, sleep quality, physical activity, time available for meal preparation, and socioeconomic status may influence both diet quality and anxiety or depressive symptoms.

Added text in Discussion, limitations: “In addition, residual confounding is an important limitation. Variables associated with both dietary habits and mental health in university students, such as sleep quality, physical activity, academic stress, and socioeconomic status, were not measured and therefore could not be included in the adjusted models. This is particularly relevant because data collection included the end-of-semester examination phase, when academic stress, sleep disruption, reduced physical activity, limited time for meal preparation, and worsening mental health symptoms may co-occur. Therefore, unmeasured academic and socioeconomic factors may partly explain the observed association between diet quality and anxiety or depression, potentially leading to underestimation or overestimation of the observed associations.”

Attachment Submitted filename: Response_to_reviewers_auresp_2.docx

# Peer review: Decision Letter 2

**DOI:** 10.1371/journal.pone.0346062.r005

**Authors:** Yordanis Enríquez Canto

3 Jun 2026

Healthier dietary habits are associated with lower depression and anxiety among medical students at a private university in Lima, Peru: A cross-sectional study

PONE-D-26-09038R2

Dear Dr. Soriano-Moreno,

We’re pleased to inform you that your manuscript has been judged scientifically suitable for publication and will be formally accepted for publication once it meets all outstanding technical requirements.

Within one week, you’ll receive an e-mail detailing the required amendments. When these have been addressed, you’ll receive a formal acceptance letter and your manuscript will be scheduled for publication.

An invoice will be generated when your article is formally accepted. Please note, if your institution has a publishing partnership with PLOS and your article meets the relevant criteria, all or part of your publication costs will be covered. Please make sure your user information is up-to-date by logging into Editorial Manager at Editorial Manager® and clicking the ‘Update My Information' link at the top of the page. For questions related to billing, please contact billing support.

If your institution or institutions have a press office, please notify them about your upcoming paper to help maximize its impact. If they’ll be preparing press materials, please inform our press team as soon as possible -- no later than 48 hours after receiving the formal acceptance. Your manuscript will remain under strict press embargo until 2 pm Eastern Time on the date of publication. For more information, please contact onepress@plos.org.

Kind regards,

Yordanis Enríquez Canto, Ph.D.

Academic Editor

PLOS One

Additional Editor Comments (optional):

Reviewers' comments:

Reviewer's Responses to Questions

**Comments to the Author**

1. If the authors have adequately addressed your comments raised in a previous round of review and you feel that this manuscript is now acceptable for publication, you may indicate that here to bypass the “Comments to the Author” section, enter your conflict of interest statement in the “Confidential to Editor” section, and submit your "Accept" recommendation.

Reviewer #2: All comments have been addressed

**********

2. Is the manuscript technically sound, and do the data support the conclusions?

The manuscript must describe a technically sound piece of scientific research with data that supports the conclusions. Experiments must have been conducted rigorously, with appropriate controls, replication, and sample sizes. The conclusions must be drawn appropriately based on the data presented.

Reviewer #2: Yes

**********

3. Has the statistical analysis been performed appropriately and rigorously?

Reviewer #2: Yes

**********

4. Have the authors made all data underlying the findings in their manuscript fully available?

The PLOS Data policy requires authors to make all data underlying the findings described in their manuscript fully available without restriction, with rare exception (please refer to the Data Availability Statement in the manuscript PDF file). The data should be provided as part of the manuscript or its supporting information, or deposited to a public repository. For example, in addition to summary statistics, the data points behind means, medians and variance measures should be available. If there are restrictions on publicly sharing data—e.g. participant privacy or use of data from a third party—those must be specified.

Reviewer #2: Yes

**********

5. Is the manuscript presented in an intelligible fashion and written in standard English?

PLOS ONE does not copyedit accepted manuscripts, so the language in submitted articles must be clear, correct, and unambiguous. Any typographical or grammatical errors should be corrected at revision, so please note any specific errors here.

Reviewer #2: Yes

**********

6. Review Comments to the Author

Please use the space provided to explain your answers to the questions above. You may also include additional comments for the author, including concerns about dual publication, research ethics, or publication ethics. (Please upload your review as an attachment if it exceeds 20,000 characters)

Reviewer #2: The authors have adequately addressed my remaining concerns. The revised manuscript now provides a stronger justification for the use of the Spanish-adapted HEI, acknowledges its lack of cultural validation in Peru, contextualises the low prevalence of "healthy" dietary habits using previous Latin American evidence, and more clearly discusses possible residual confounding related to the examination period, academic stress, socioeconomic status and other unmeasured factors. The authors also assessed the linearity assumption before modelling the HEI score as a continuous exposure. I have no further substantive concerns.

**********

7. PLOS authors have the option to publish the peer review history of their article (what does this mean?). If published, this will include your full peer review and any attached files.

If you choose “no”, your identity will remain anonymous but your review may still be made public.

**Do you want your identity to be public for this peer review?**   For information about this choice, including consent withdrawal, please see our Privacy Policy.

Reviewer #2: No

**********

# Editor report: Acceptance letter

**DOI:** 10.1371/journal.pone.0346062.r006

**Authors:** Yordanis Enríquez Canto

PONE-D-26-09038R2

PLOS One

Dear Dr. Soriano-Moreno,

I'm pleased to inform you that your manuscript has been deemed suitable for publication in PLOS One. Congratulations! Your manuscript is now being handed over to our production team.

At this stage, our production department will prepare your paper for publication. This includes ensuring the following:

* All references, tables, and figures are properly cited

* All relevant supporting information is included in the manuscript submission,

* There are no issues that prevent the paper from being properly typeset

You will receive further instructions from the production team, including instructions on how to review your proof when it is ready. Please keep in mind that we are working through a large volume of accepted articles, so please give us a few days to review your paper and let you know the next and final steps.

Lastly, if your institution or institutions have a press office, please let them know about your upcoming paper now to help maximize its impact. If they'll be preparing press materials, please inform our press team within the next 48 hours. Your manuscript will remain under strict press embargo until 2 pm Eastern Time on the date of publication. For more information, please contact onepress@plos.org.

You will receive an invoice from PLOS for your publication fee after your manuscript has reached the completed accept phase. If you receive an email requesting payment before acceptance or for any other service, this may be a phishing scheme. Learn how to identify phishing emails and protect your accounts at https://explore.plos.org/phishing.

If we can help with anything else, please email us at customercare@plos.org.

Thank you for submitting your work to PLOS ONE and supporting open access.

Kind regards,

PLOS ONE Editorial Office Staff

on behalf of

Prof. Yordanis Enríquez Canto

Academic Editor

PLOS One
